# Pharmacists delivering hypertension care services: a systematic review and meta-analysis of randomized controlled trials

**DOI:** 10.3389/fcvm.2025.1477729

**Published:** 2025-03-14

**Authors:** Viktoria Gastens, Stefano Tancredi, Blanche Kiszio, Cinzia Del Giovane, Ross T. Tsuyuki, Gilles Paradis, Arnaud Chiolero, Valérie Santschi

**Affiliations:** ^1^ Population Health Laboratory (#PopHealthLab), University of Fribourg, Fribourg, Switzerland; ^2^ La Source, School of Nursing Sciences, HES-SO University of Applied Sciences and Arts Western Switzerland, Lausanne, Switzerland; ^3^ Department of Medical and Surgical Sciences for Children and Adults, University-Hospital of Modena and Reggio Emilia, Modena, Italy; ^4^ EPICORE, Department of Medicine, Division of Cardiology, Faculty of Medicine and Dentistry, University of Alberta, Edmonton, AB, Canada; ^5^ School of Population and Global Health, McGill University, Montreal, QC, Canada

**Keywords:** hypertension, blood pressure, pharmacists, pharmaceutical care, systematic review, meta-analysis

## Abstract

**Background:**

Community-based models of care with the involvement of pharmacists and other nonphysician healthcare professionals can help improve blood pressure (BP) control. We aimed to synthesize the evidence of effectiveness of pharmacist interventions on BP among patients with hypertension.

**Methods:**

We performed systematic searches to identify randomized controlled trials (RCTs) assessing the effect of pharmacist interventions on BP among outpatients (latest search, March 2024). The effect on systolic and diastolic BP change or BP control were pooled using random effects model. Subgroup analysis for the types of pharmacist interventions and healthcare settings were performed. The risk of bias was assessed using the Cochrane Risk of Bias Tool 2. The protocol was registered in PROSPERO (CRD42021279751) and published in an open-access peer-reviewed journal.

**Results:**

Out of 2,330 study records identified in 7 electronic databases, a total of 95 RCTs, with 31,168 participants (control 16,157, intervention 15,011), were included. The intervention was led by the pharmacist in 75% of the studies and in collaboration with other healthcare providers in 25%. Pharmacist interventions included patient education in 88%, feedback to healthcare providers in 49%, and patient reminders in 24% of the studies. Systolic and diastolic BP were reduced after pharmacist intervention by −5.3 mmHg (95% CI: −6.3 to −4.4; *I*^2^ = 86%) and −2.3 mmHg (95% CI: −2.9 to −1.8; *I*^2^ = 75%), respectively. The reduction of systolic BP tended to be larger if the intervention was collaborative, conducted in outpatient clinics, based on healthcare provider education, or through healthcare provider feedback. Analyses restricted to relatively large or high-quality studies yielded similar estimates, with lower between-studies heterogeneity.

**Conclusion:**

Pharmacist care for patients with hypertension consistently improves BP across various settings and interventions. Pharmacist care is one key element of the solution to the global burden of hypertension and cardiovascular diseases.

**PROSPERO registration number:**

CRD42021279751.

## Introduction

High blood pressure (BP) is the leading contributor to global mortality due to its impact on cardiovascular diseases (CVD), particularly coronary heart disease and stroke (1). A reduction of 5 mmHg in systolic BP can decrease the relative risk of major cardiovascular events by approximately 10% (2) and lowering BP levels is therefore crucial in preventing CVD and reducing their burden. However, many patients are not diagnosed and, if diagnosed, are poorly controlled and do not reach target BP levels; this might be due to limited access to healthcare or lifestyle counseling, clinical inertia, or non-adherence to medication (3).

To improve BP control, one strategy is to involve non-physician healthcare professionals such as pharmacists, nurses, or community health workers in the management of hypertension. The US Community Preventive Services Task Force, and more recently the American College of Cardiology, the American Heart Association, and the European Societies of Cardiology and Hypertension all recommend team-based care in hypertension management, with the involvement of pharmacists (4–7). Indeed, pharmacists have a pivotal role as highly accessible healthcare providers and experts in medication management. For instance, they can provide patient education, e.g., to improve health behaviours, and help patients adhere to medications. Community pharmacists are therefore a logical choice and a valuable asset to contribute to BP management.

Several meta-analyses of randomized trials have shown that pharmacist interventions, either pharmacist directed or in collaboration with other healthcare professionals, can help decrease BP (8, 9). However, the effects of the interventions on BP vary widely, from very large to modest or no effect, and the reasons for this heterogeneity are not entirely clear (8). Even when an effect is observed, the heterogeneity of the interventions can limit the implementation of guidelines and recommendations. Indeed, team-based care interventions are typically multi-component, and it can be challenging to assess the effect of each component. Additionally, the interventions can differ in terms of intensity, target population, settings or the number and type of professionals involved. It is therefore key to try to identify what works best, including duration or the intensity of intervention, patient characteristics or different patient care settings.

We therefore aimed to systematically review, synthesize, and update the evidence of the effect of pharmacist in delivering hypertension care services through directed care or in collaboration with other healthcare professionals on BP level among hypertensive patients. We also evaluated the heterogeneity in the effect of these interventions, specifically to determine which interventions are most effective in different healthcare contexts.

## Methods

We followed the Cochrane Collaboration and Center for Reviews and Dissemination guidance methods for conducting and reporting systematic reviews and meta-analyses (10, 11) and report the results of this review according to the Preferred Reporting Items for Systematic Reviews and Meta-Analyses statement (PRISMA) (12). The protocol for this systematic review was registered on the International Prospective Register of Systematic Reviews (PROSPERO) database (CRD42021279751) and published in an open-access peer-reviewed journal (13).

### Eligibility criteria

Inclusion and exclusion study criteria were based on specific (1) study designs, (2) settings, (3) participants, (4) interventions, (5) comparators, and (6) outcomes. Randomized controlled trials (RCTs), cluster RCTs, and cross-over RCTs were eligible. Case reports, case series, non-randomized evaluations, reviews, meta-analyses, conference proceedings, policy papers, study protocols, and expert opinions were excluded. Studies based in a community/ambulatory care setting were included. Studies were considered if they included adult outpatients (18 years or over) with a diagnosis of hypertension, treated or not treated, and if they evaluated the effect of pharmacist interventions—directed or in collaborative care—in outpatients with hypertension compared to usual care. We distinguished between outpatient clinics (hospital or medical facility without overnight stay) and community care (e.g., at a community pharmacy or general practitioner). We included interventions that were delivered by a pharmacist directed or in collaborative care. Outcomes of interest were the mean difference in BP change, or BP control (BP below a predefined target level) at follow-up. We considered all publications in English, French, and German and searched all databases from inception to the date of search.

### Information sources

We searched the following electronic databases: MEDLINE (Ovid) (from 1946 on), Excerpta Medica database (Embase) (from 1947 on), Cochrane Central Register of Controlled Trials (CENTRAL) (from 1947 on), Cochrane Database of Systematic Reviews (CDSR) (from 1995 on), CINAHL (EBSCO) (from 1937 on), Web of Science (from 1900 on), JBI EBP Database (Ovid) (from 1998 on), and Tripdatabase (from 1997 on). The search for unpublished studies included the Grey Literature Report (New York Academy of Medicine, http://www.greylit.org). The search was conducted on 26.03.2024.

### Search strategy

The specific search strategies (Supplementary Table S1) were developed by an experienced medical librarian in systematic review searching (BK) in consultation with the project team. They were constructed to include the two main concepts of this systematic review: “hypertension” and “pharmacist intervention”. A three-step search strategy was used in this review. First, an initial limited search of MEDLINE (Ovid) was undertaken using the search terms “Pharmacist intervention”, “Pharmacists”, “Pharmaceutical Services”, “Pharmacy Service, Hospital”, “Pharmacies”, “Pharmacy”, “Hypertension”, “Blood pressure”. Second, an analysis of the text words contained in each article's title, abstract, and index terms was undertaken to expand the list of search terms. Based on the results of this analysis, a more thorough search was conducted in the chosen databases. The search strategy for MEDLINE was created first and was then adapted for each database, including all identified keywords and index terms. Third, the reference lists of all included studies selected for critical appraisal were searched by hand and cited reference searches for all included studies were conducted in Web of Science to find any additional studies not identified during the initial search processes. The methodology search filter to limit retrieval to appropriate study designs, a modified version of the Cochrane Highly Sensitive Search Strategy, was used to identify randomized trials (10).

### Selection process

Study records retrieved by electronic searching were uploaded to the systematic review management software Covidence (14). After the removal of duplicates, titles, and abstracts were independently screened by two reviewers (VG and ST) for inclusion. The reviewers indicated whether a citation is potentially relevant (met inclusion criteria), is clearly not relevant (met exclusion criteria), or if the information is insufficient to make a judgment. We obtained full-text publications for all titles/abstracts that appear to meet inclusion criteria or where there is any uncertainty. Full-text publications were independently examined by two reviewers (VG and ST) to select studies for inclusion. Reasons for exclusion of ineligible studies were recorded. Any disagreement was resolved through discussion and, if required, by consulting a third review author (AC or VS). The selection process was recorded in detail in a Preferred Reporting Items for Systematic Reviews and Meta-Analyses (PRISMA) flow diagram (12).

### Data collection process

Data extraction was conducted using a prespecified data extraction template in the systematic review management software Covidence (14). Data was independently extracted by two reviewers (VG and ST) from each eligible study. Using a structured data collection form, these two reviewers independently extracted the data listed in Supplementary Table S2.

Primary outcome data to extract was the mean difference between intervention and control group in systolic and diastolic BP change from baseline to follow-up and the corresponding standard error (continuous outcome). If not reported in studies, the mean difference in BP change and standard error was calculated from reported information (BP change per study group, BP at baseline, BP at follow-up, and standard deviation, corresponding confidence intervals or *p* values of subgroup analyses). If no BP change or BP baseline information was available, the mean difference between intervention and control group at follow-up was used (15). For BP control (dichotomous, secondary outcome), we extracted the proportion of participants reaching a pre-defined BP target level. The target BP could differ from one study to the other.

We classified pharmacist interventions using the following pre-defined categories: (1) pharmacist-directed care (pharmacist initiating and managing care) and (2) pharmacist collaborative care (pharmacist collaborating in interventions conducted by a multidisciplinary healthcare team) (16). Based on the Cochrane Effective Practice and Organization of Care (EPOC) taxonomy interventions, we categorized pharmacist interventions by different target levels (patient, healthcare provider) and types (educational approach, e.g., targeted toward patients to improve their lifestyle; feedback, e.g., to healthcare providers to adapt medication; use of reminder tools, e.g., drug adherence aids) (17).

### Risk of bias assessment

Two reviewers (VG and ST) independently assessed the risk of bias for each study using the “Cochrane Risk of Bias Tool” for randomized trials (RoB 2) (18). This tool assesses the risk of bias according to the following domains: 1. Randomization process; 2. Effect of assignment to intervention; 3. Missing outcome data; 4. Measurement of the outcome; 5. Selection of the reported result. The risk of bias assessment for cluster-randomized trials and randomized crossover trials was performed with the specific RoB 2 tools for these study designs (18).

We classified the risk of bias for each domain as either “Low risk”, “Some concerns” or “High risk” and provided information from each study together with the reasons for our evaluation (10, 18). Given the type of RCTs included in our review, blinding the participants and the research teams was usually not feasible; only the outcome assessment could be blinded. The quality of BP measurement was also systematically assessed along three criteria: (1) use of clinically validated BP measurement devices; (2) training of outcome assessor; (3) measurement of BP out of the office. In accordance with the revised Cochrane risk of bias tool for randomized trials, RoB2, we derived an overall study risk of bias as follows: “Low risk” with all domains at low risk of bias, “Some concerns” with at least one domain of some concern, “High risk” with high risk of bias in at least one domain or with some concerns for two or more domains (18). We resolved any disagreement in quality assessment through discussions and involvement of an arbitrator (AC or VS) where necessary.

### Effect measures

For the continuous outcome, we used the mean difference in BP change between groups whenever available. When this measure was not reported, we used the mean difference in BP between groups at the end of follow-up (15). Calculations for the between-group standard error were based on calculations provided in the Cochrane Handbook (10). The pooled effect was calculated as weighted mean differences in BP between intervention and usual care groups, with 95% confidence intervals (CI). For the dichotomous outcome of BP control, we estimated the pooled relative risk (RR) comparing intervention vs. usual care groups, with 95% CI.

### Statistical analyses

All analyses were conducted with R 4.2.2 (The R Foundation) using the meta software package (19). For the secondary outcome BP control, we calculated the relative risk as the ratio in the proportion of BP control (%) at follow-up in pharmacist intervention group vs. usual care group. A random effects model was used to estimate the pooled effects and results displayed in forest plots. Between-studies heterogeneity was quantified using the *I*^2^ statistic. To explore the possible sources of heterogeneity, we conducted subgroup analyses by categories of selected study characteristics: region, setting, type of pharmacist care, healthcare team composition, intervention characteristics. To assess the robustness of our results, we performed sensitivity analyses: among studies at high quality and with larger study size. Publication bias was assessed by visual inspection of funnel plots, and funnel plot asymmetry was examined using the Egger test (10, 20). The Grading of Recommendations Assessment, Development and Evaluation (GRADE) framework was applied to assess the strength of the body of evidence for this systematic review (21). Domains used to assess the certainty of the evidence included risk of bias, inconsistency, indirectness, imprecision, and publication bias. The certainty of evidence was assessed for systolic BP both by including all studies and by excluding studies with a high risk of bias.

## Results

### Study selection and characteristics

**Figure 1:**
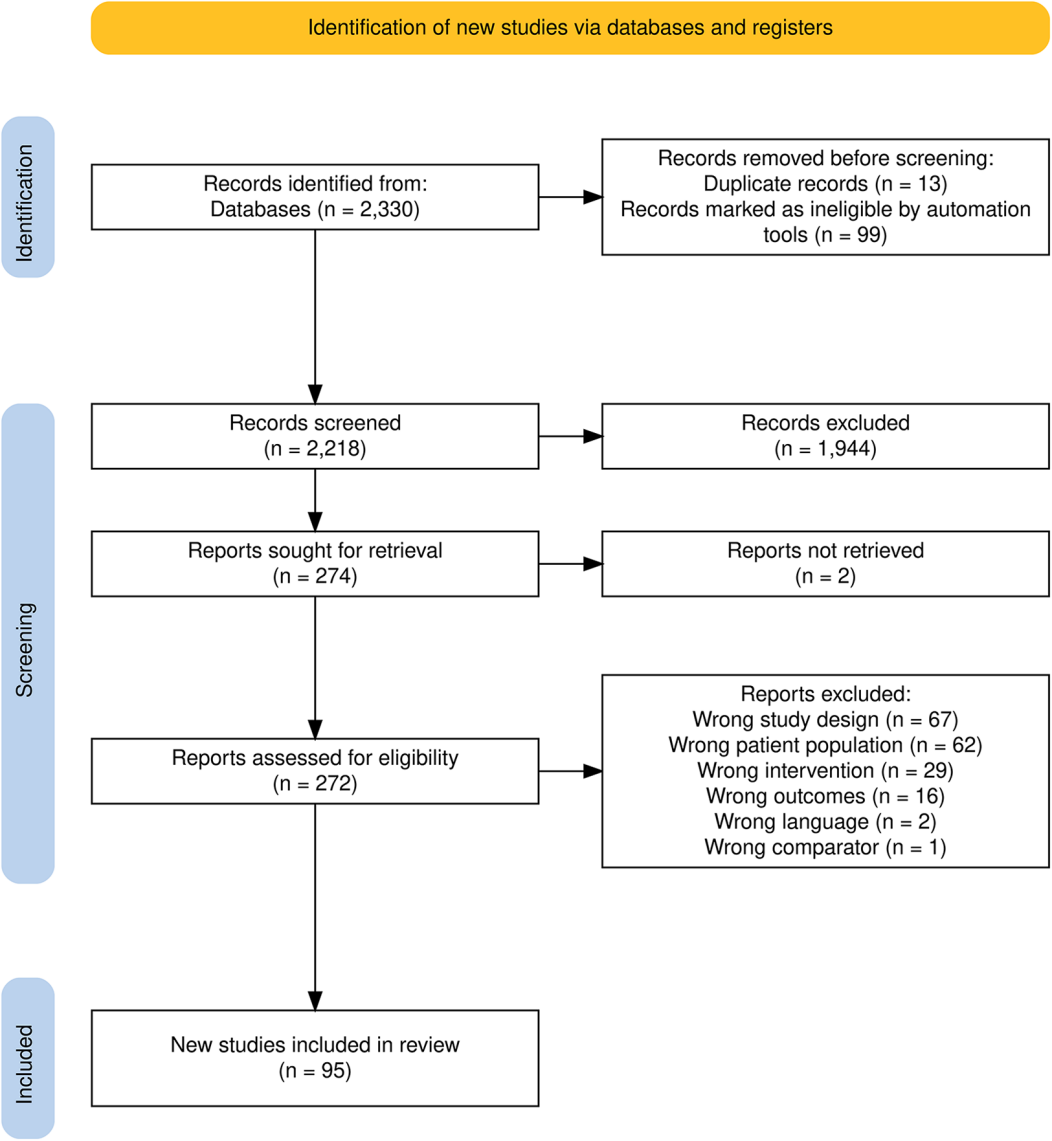
PRISMA 2020 flow diagram of studies identified, included, and excluded in the systematic review (22).

A total of 2,330 study records were identified by electronic database searching (Supplementary Table S1) and loaded to the systematic review management software Covidence (14). After removal of duplicates, 2,218 studies were independently screened based on title and abstract by two authors, and 274 full texts were evaluated for eligibility (Figure 1). A total of 95 studies, all published in the English language, were included in our review (23–114), including 31,168 participants (control 16,157, intervention 15,011). The characteristics of each study are presented in Supplementary Table S3. Reasons for exclusion of studies (*n* = 179) were most commonly not appropriate study design (*n* = 67), not appropriate patient population (*n* = 62) and not appropriate intervention (*n* = 29) (115–117).

A summary of studies and patient characteristics is shown in Table 1. Included studies were published between 1973 and 2023 and conducted in different regions (North America: *n* = 47, Europe: *n* = 17, other: *n* = 31). The characteristics of the pharmacist interventions are summarized in Table 2. In 75% of the studies, the intervention was led by the pharmacist and in 25% in collaboration with other healthcare providers. In the 24 pharmacist collaborative care studies, the team composition was pharmacist/physician (*N* = 12); pharmacist/nurse/physician (*N* = 4); pharmacist/nurse/physician/diabetes educator (*N* = 2), or pharmacist/nurse (*N* = 6). Interventions targeted patients in 93% and healthcare providers in 61% of the studies. The types of intervention comprised patient education in 88%, healthcare provider education in 14%, feedback to healthcare providers (e.g., medication change) in 49%, and patient reminders (e.g., drug adherence aids) in 24% of the studies, respectively. Feedback to healthcare providers included medication change, either after independent pharmacist prescribing or recommendations to physicians. Further details of study and pharmacist intervention characteristics for each included study are displayed in Supplementary Table S3.

**Table 1:** Summary of study and patient characteristics of the included studies.

Study characteristics	*N* = 95
Region	
Pan-American	47 (49%)
European	17 (18%)
Western Pacific	12 (13%)
South-East Asian	10 (11%)
Eastern Mediterranean	6 (6%)
African	3 (3%)
Setting	
Outpatient clinics	62 (65%)
Community pharmacies	31 (33%)
Duration of follow-up in months	
Mean (min, max)	8 (1.5, 48)
Outcome	
Systolic BP change	48 (51%)
Systolic BP at follow-up	28 (29%)
BP control	62 (65%)
Patient characteristics	
Mean age	
Mean (min, max)	61 (39, 80)

**Table 2:** Summary of pharmacist intervention characteristics of the included studies.

Intervention characteristics	*N* = 95
Pharmacist care	
Pharmacist-directed	71 (75%)
Pharmacist-collaborative	24 (25%)
Healthcare providers involved	
Physician	34 (36%)
Nurse	14 (15%)
Other	3 (3%)
Type of EPOC intervention	
- At patient level	
Education (e.g., lifestyle counselling)	84 (88%)
Reminder (e.g., drug adherence aids)	23 (24%)
- At healthcare provider level	
Educational material (e.g., leaflets)	11 (12%)
Educational meeting (e.g., workshops)	9 (9%)
Feedback (e.g., medication change)	47 (49%)
Reminder (e.g., software tool)	1 (1%)
Number of interventions	
Mean (min, max)	3.8 (1, 6)
Duration of interventions, in months	
Mean (min, max)	6.4 (1, 12)
Frequency of interventions	
Once a month or more frequently	21 (22%)
Less than once a month	10 (11%)
Irregular or not clearly specified	64 (67%)
Duration of each intervention session, in minutes	
Mean (min, max)	24 (5, 37.5)

Results of the risk of bias assessment for each included study and the five domains (randomization process; deviations from intended interventions; missing outcome data; measurement of the outcome; selection of the reported result) are shown in Supplementary Figure S1. Overall, the quality of the studies was relatively low. Some 54 studies (57%) were at high risk of bias, 41 studies (43%) raised some concerns, and no study was assessed at low risk. The method of blood pressure measurement was assessed as appropriate or probably appropriate in 66 studies (69%), inappropriate or probably inappropriate in 5 studies (5%), and missing information in 24 studies (25%).

### Effect on blood pressure

**Figure 2:**
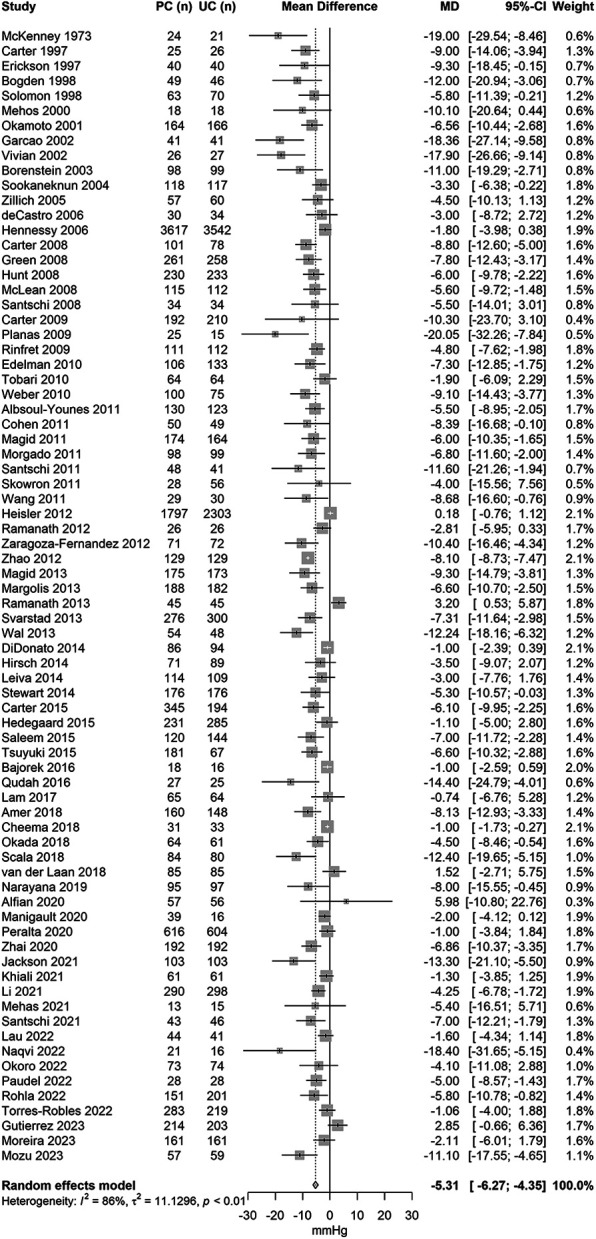
Forest plot of the mean difference in systolic blood pressure change between pharmacist and usual care group sorted by year of publication.

**Figure 3:**
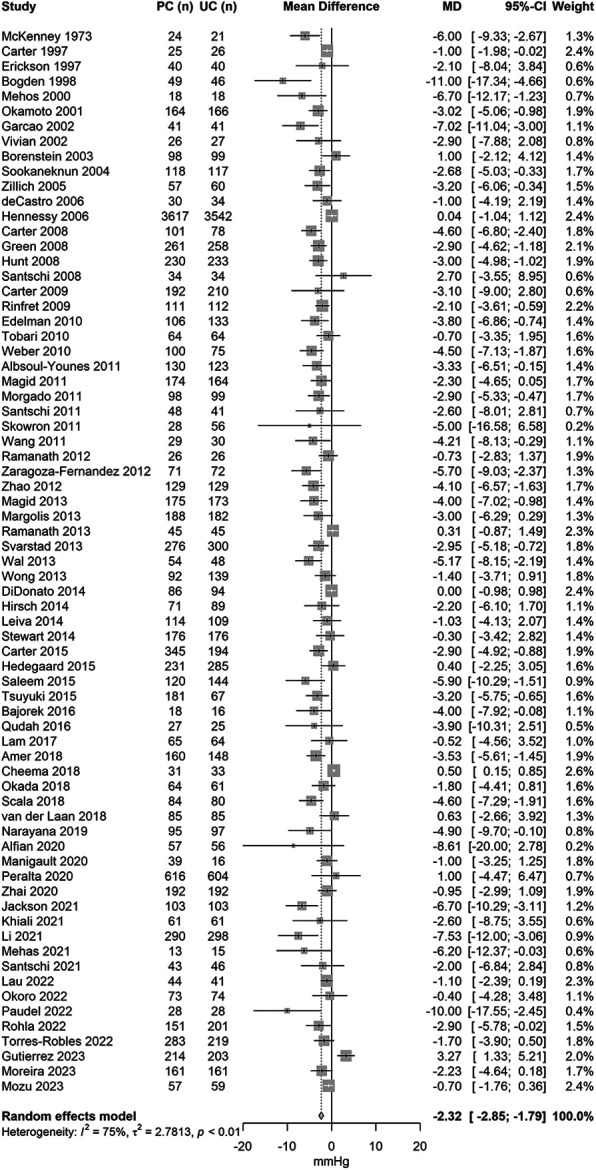
Forest plot of the mean difference in diastolic blood pressure change between pharmacist and usual care by year of publication.

The forest plots of the mean change in systolic and diastolic BP change are displayed in Figure 2 (76 studies, *N* = 27,057) and Figure 3 (71 studies, *N* = 22,652). The pooled results showed that pharmacist care services alone or in collaboration with other healthcare professionals reduced systolic/diastolic BP by −5.3 (95% CI: −6.3 to 4.4)/−2.3 (95% CI: −2.9 to −1.8) mmHg. There was a large between-study heterogeneity, with an *I*^2^ of 86% for systolic BP and of 75% for diastolic BP.

Results of subgroup analyses are displayed in Table 3. Overall, there were no major differences in the effect on BP according to study characteristics or types of intervention. No difference in BP reduction was observed by region of study and pharmacist directed/collaborative care. The effects on systolic BP tended to be slightly larger if the intervention was in an outpatient clinic [−6.0 mmHg (95% CI: −7.2 to −4.8)], collaborative [−6.2 mmHg (95% CI: −7.6 to −4.9)], based on healthcare provider education [−6.1 mmHg (95% CI: −9.2 to −3.0)], or through healthcare provider feedback [−6.1 mmHg (95% CI: −7.2 to −4.9)].

**Table 3:** Subgroup analyses for the difference in systolic and diastolic blood pressure (BP) with pharmacist care compared with usual care group according to study characteristics.

Characteristic	Systolic BP (mmHg)			Diastolic BP (mmHg)		
	N	Mean difference (95% CI)	P	N	Mean difference (95% CI)	*p*
Overall	76	−5.3 (−6.3; −4.4)	<0.01*.	71	−2.3 (−2.9; −1.8)	<0.01*
Region						
Pan-American	39	−5.8 (−7.1; −4.5)	0.46**	33	−2.4 (−3.0; −1.7)	0.57**
European	13	−5.0 (−7.7; −2.3)		13	−2.0 (−3.5; −0.6)	
Western Pacific	9	−4.0 (−6.5; −1.5)		10	−1.8 (−3.6; −0.1)	
South-East Asian	7	−3.7 (−7.7; 0.3)		7	−3.1 (−5.4; −0.7)	
Eastern Mediterranean	5	−5.9 (−9.3; −2.5)		5	−3.7 (−5.3; −2.2)	
African	3	−9.4 (−14.7; −4.1)		3	−2.4 (−6.3; 1.4)	
Setting						
Outpatient	49	−6.0 (−7.2; −4.8)	0.02**	46	−2.6 (−3.2; −2.0)	0.21**
Community	25	−4.1 (−5.7; −2.5)		23	−1.9 (−3.0; −0.9)	
Pharmacist care						
Pharmacist-collaborative	22	−6.2 (−7.6; −4.8)	0.15**	20	−2.7 (−3.5; −2.0)	0.33**
Pharmacist-led care	54	−4.9 (−6.1; −3.7)		51	−2.2 (−2.9; −1.6)	
Team						
Physician involved						
Yes	32	−6.3 (−7.6; −4.9)	0.09**	31	−2.6 (−3.2; −1.9)	0.46**
No	44	−4.7 (−5.9; −3.4)		40	−2.2 (−2.9; −1.4)	
Nurse involved						
Yes	11	−6.3 (−7.8; −4.7)	0.22**	8	−1.8 (−2.8; −0.9)	0.33**
No	65	−5.1 (−6.2; −4.1)		63	−2.4 (−3.0; −1.8)	
Intervention (EPOC)						
Patient education						
Yes	67	−5.2 (−6.2; −4.1)	0.24**	62	−2.2 (−2.8; −1.6)	0.14**
No	9	−6.6 (−8.6; −4.5)		9	−3.2 (−4.3; −2.1)	
Patient reminder						
Yes	20	−4.3 (−6.0; −2.6)	0.20**	19	−1.8 (−3.0; −0.6)	0.29**
No	56	−5.7 (−6.8; −4.5)		52	−2.5 (−3.1; −1.9)	
Healthcare provider education						
Yes	10	−6.1 (−9.2; −3.0)	0.62**	9	−2.9 (−4.5; −1.3)	0.49**
No	66	−5.2 (−6.2; −4.2)		62	−2.3 (−2.8; −1.7)	
Healthcare provider feedback						
Yes	41	−6.1 (−7.2; −4.9)	0.08**	38	−2.6 (−3.2; −2.0)	0.23**
No	35	−4.4 (−5.8; −2.9)		33	−2.0 (−2.8; −1.1)	
Frequency of interventions						
Once a month or more	15	−7.4 (−9.9; −4.9)	0.16**	14	−2.3 (−3.5; −1.1)	0.97**
Less than once a month	8	−4.6 (−6.5; −2.7)		8	−2.2 (−3.3; −1.0)	
Irregular or not specified	53	−4.9 (−6.0; −3.8)		49	−2.3 (−3.0; 1.7)	

* *p* value for the between-group (intervention vs. usual care) difference.

** *p*-value for the subgroup (by characteristics) differences.

Sensitivity analysis restricted to studies at relatively low risk of bias yielded similar results (mean difference of −4.8 mmHg (95% CI: −5.9 to −3.7 mmHg; *I*^2^ = 87%) for systolic BP (*N* = 39, including 21,339 participants) and −2.3 mmHg (95% CI: −3.0 to −1.6 mmHg; *I*^2^ = 63%) for diastolic BP (*N* = 35, including 16,780 participants), respectively), but the between-study heterogeneity was much smaller. The forest plots of the mean change in systolic and diastolic BP restricted to studies with a relatively low risk of bias are displayed in Supplementary Figures S2, S3. Sensitivity analysis excluding studies of small size (less than 50 total participants) resulted in a mean difference between pharmacist intervention and usual care of −5.1 mmHg (95% CI: −6.1 to −4.2 mmHg; *I*^2^ = 87%) for systolic BP (*N* = 70, 26,837 participants) and −2.2 mmHg (95% CI: −2.7 to −1.7 mmHg; *I*^2^ = 74%) for diastolic BP (*N* = 65, 21,766 participants). These differences were of the same magnitude compared with the differences observed when all studies were included.

The secondary outcome BP control at follow-up was reported in 62 studies (*N* = 22,657). At baseline, the median proportion of participants with controlled BP was 0% (25th to 75th percentile: 0.0%–37.4%) in the usual care group and 0% (25th to 75th percentile: 0.0%–35.4%) in the pharmacist care group. At follow-up, the median proportion of participants with controlled BP was 40.8% (25th to 75th percentile: 29.9%–57.0%) in the usual care group and 61.2% (25th to 75th percentile: 46.1%–69.6%) in the pharmacist care group. The mean difference in the change from baseline to follow-up in the proportion of participants with controlled BP was +17.3% (95% CI: + 13.3% to +21.2%) between pharmacist care and usual care group. The pooled results showed a relative risk for BP control after pharmacist intervention of 1.37 (95% CI: 1.27 to 1.47; *I*^2^ = 80%; Supplementary Figure S4).

### Reporting bias and GRADE assessment

Potential publication bias was observed for both systolic and diastolic BP as suggested by the asymmetry of the funnel plots (Supplementary Figure S5), with Egger test *p*-value of 0.03 for systolic BP and <0.01 for diastolic BP. When limited to relatively high-quality studies, the funnel plot asymmetry disappeared (Supplementary Figure S6**)**, with Egger test *p*-values of 0.65 for systolic BP and 0.07 for diastolic BP. The GRADE framework was applied to assess the strength of the body of evidence for this systematic review (Supplementary Table S4) (21). The certainty of evidence for systolic BP was assessed as low when including all studies (the certainty was downgraded due to study limitations and publication bias) and moderate when excluding studies with a high risk of bias (downgrading for study limitation). This means that the true effect is likely to be close to effect estimate, but the possibility that it is substantially different exists.

## Discussion

Our systematic review and meta-analysis of almost 100 studies confirmed a clinically and statistically significant reduction in BP with pharmacist care services. The BP difference as a result of pharmacists' interventions, was a 5.3 mmHg (95% CI: −6.3 to −4.4; *I*^2^ = 86%) reduction in systolic BP and a 2.3 mmHg (95% CI: −2.9 to −1.8; *I*^2^ = 75%) reduction in diastolic BP. There was also a large effect on BP control. Similar estimates were found when excluding studies at high risk of bias. Our results showed that different types of pharmacists' interventions reduce BP, improve BP control, and support the involvement of pharmacists in BP management. We found that the effects on systolic BP tended to be larger if the intervention was collaborative, based on healthcare provider education, or through healthcare provider feedback. Moreover, we observed a greater improvement in systolic BP in outpatient clinics compared to community pharmacies.

The BP reduction estimates found in this systematic review are comparable to estimates found in previous systematic reviews (8, 118). In a systematic review conducted in 2014 by our team, including 39 RCTs for systolic BP and 36 for diastolic BP, we found a BP reduction of −7.6 mm Hg (95% CI: −9.0 to −6.3 mm Hg) and −3.9 mm Hg (95% CI: −5.1 to −2.8 mm Hg) for SBP and DBP respectively (8). Similar estimates were reported in other studies; Chisholm-Burns et al. found a mean difference between the pharmacist group and the comparison group of −7.8 mm Hg (95% CI: −9.7 to −5.8) in systolic BP, and −2.9 mm Hg (95% CI: −3.8 to −2.0) in diastolic BP (118). Other systematic reviews only assessed specific setting or interventions. For instance, Baral et al. found that the addition of pharmacist-led home BP telemonitoring to usual care leads to a significant decrease in systolic BP (−8.1 mm Hg; 95% CI: −11.2 to −5.0 mm Hg) and diastolic BP (−4.2 mm Hg; 95% CI: −5.6 to −2.8 mm Hg) compared to usual care (119). Our systematic review provides the highest level of evidence to develop public policies to implement pharmacist care in hypertension. This transition from evidence to real-life policies should be supported by implementation studies (120).

The most common interventions, whether directed by the pharmacist or in collaboration with other healthcare professionals, included educating patients (about medications, lifestyle, or compliance), providing feedback to physicians (identifying medication-related problems and suggesting adjustments), and using reminders for medication management. Physician and nurse collaboration were the most common choice for healthcare teams in collaborative interventions. However, analyses aimed at comparing the effectiveness of specific intervention or type of care did not yield significant differences across intervention types. This might be attributed to the fact that multiple types of interventions effectively contribute to reduce BP, or to the challenge of disentangling the multifaceted and heterogeneous nature of these interventions, usually consisting of several components. One possible approach to gain a deeper understanding of the role of each specific intervention type could be to assess the comparative effectiveness of interventions using network meta-analysis (121). As for the effectiveness of pharmacist interventions in different settings, we observed a slightly greater reduction in BP for interventions conducted in outpatient clinics compared to those in community pharmacies. This could be due to several factors. One hypothesis is that patients in outpatient clinics may be more committed to improving their health status, or the consistent contact with healthcare professionals in outpatient clinics could lead to better adherence to recommendations, including medication and lifestyle changes. Furthermore, patients in outpatient clinics could benefit from more extended and focused consultations with pharmacists and other healthcare providers. This additional time allows for more in-depth education and counselling (122). Outpatient clinics are usually connected to larger facilities such as teaching hospitals or university hospitals with more resources than community pharmacies and usually have fixed patient appointments instead of walk-in services.

When interpreting the results of this study, several limitations should be considered. Firstly, trials for pharmacists' intervention face practical challenges in blinding both participants and researchers, increasing risk of bias. Hence, we found no study at low risk of bias, and many included studies were considered at high risk. This limitation also affected the GRADE assessment of the certainty of the evidence, resulting in a low-certainty rating. However, a recent review concluded that it is rare to fulfil the criteria for high-quality evidence with GRADE (123). Low-quality trials are unfortunately a major and pervasive issue in biomedical and clinical research (124). Furthermore, our analyses indicated potential publication bias, i.e., a preference for publishing studies with favourable results of pharmacist interventions over those reporting negative outcomes. This bias disappeared when assessing studies at moderate risk of bias only, or when evaluating diastolic BP. Moreover, we observed high heterogeneity in the included studies. This high heterogeneity may be explained by the large variation in interventions and settings. Due to this complexity and multiple elements of both interventions and usual care, a sharp contrast between the different types of interventions was difficult to show. Despite the very large number of studies, identifying which elements of pharmacists' interventions were the most effective remained difficult and disputable. The assessment of complex healthcare interventions (125, 126), is a standard problem which can limit the ability to make strong statements on what works best. Additional limitations include the absence of specific information regarding usual care in numerous studies and the relatively low number of studies with a long follow up, which makes it difficult to understand the long-term impact of pharmacists' interventions on blood pressure management. We also lack studies evaluating the effect of pharmacist interventions on the occurrence of blood pressure related cardiovascular diseases or mortality. Lastly, there is a need for a cost-effectiveness analysis of these interventions, which would assist in identifying the most preferable intervention for implementation (127). This study's strengths lie in its comprehensive and systematic approach and the substantial number of studies included, conducted across several world regions. Moreover, it adheres to established guidelines, such as the Cochrane guidelines for Systematic Reviews and the PRISMA statement (10, 12).

In conclusion, our systematic review of almost 100 RCTs provides the highest level of evidence for the impact of pharmacist care in hypertension. This evidence should inform changes to the delivery of care in hypertension and would help to address the approximately 50% of patients with uncontrolled hypertension. New policies and research should focus on the implementation of these interventions in real-life settings with rigorously conducted evaluation and monitoring studies including e.g., patient-reported outcomes and cost-effectiveness analyses (128).

## Data Availability

The original contributions presented in the study are included in the article/Supplementary Material, further inquiries can be directed to the corresponding author.
