# Supplementary material for: Pharmacists delivering hypertension care services: a systematic review and meta-analysis of randomized controlled trials
Source: Front Cardiovasc Med. 2025 Mar 14;12:1477729. doi: 10.3389/fcvm.2025.1477729 (PMC11949927; doi:10.3389/fcvm.2025.1477729)
Supplement: Supplementary file 1 [file Datasheet1.docx]

**SUPPLEMENTAL MATERIAL**

Pharmacists delivering hypertension care services: a systematic review and meta-analysis of randomized controlled trials

Viktoria Gastens, Stefano Tancredi, Blanche Kiszio, Cinzia Del Giovane, Ross T. Tsuyuki, Gilles Paradis, Arnaud Chiolero, Valérie Santschi

**Supplemental Table S1.** Full search strategies for all databases, registers, and websites, including any filters and limits used.

**Supplemental Table S2.** Independently extracted data during the systematic review process.

**Supplemental Table S3.** Study and pharmacist intervention characteristics for each included study.

**Supplemental Table S4.** Grading of Recommendations Assessment, Development and Evaluation (GRADE) assessment of the evidence for the effect of pharmacist interventions on systolic blood pressure (BP).

**Supplemental Figure S1.** Results of the risk of bias assessment using the revised Cochrane risk of bias (RoB 2) tool and visualised with the robvis tool.

**Supplemental Figure S2.** Sensitivity analysis limited to relatively high-quality studies. Forest plot of the mean difference between pharmacist and usual care group in systolic blood pressure sorted by year of publication.

**Supplemental Figure S3.** Sensitivity analysis of relatively high-quality studies. Forest plot of the mean difference between pharmacist and usual care group in diastolic blood pressure sorted by year of publication.

**Supplemental Figure S4.** Forest plot of the relative risk between pharmacist and usual care group in blood pressure control sorted by year of publication.

**Supplemental Figure S5.** Funnel plots to assess publication bias for systolic blood pressure (left panel) and diastolic blood pressure (right panel).

**Supplemental Figure S6.** Funnel plots to assess publication bias, excluding studies at high risk-of-bias for systolic blood pressure (left panel) and diastolic blood pressure (right panel).

**Supplemental Table S1.** Full search strategies for all databases, registers, and websites, including any filters and limits used.

| Ovid Medline Search Strategy | 1. Pharmacists/ or Community Pharmacy Services/ or Pharmaceutical Services/ or Pharmaceutical Services, Online/ or Pharmacy Service, Hospital/ or Pharmacies/ or Pharmacy/ or Evidence-Based Pharmacy Practice/ or Pharmacy research/ or Drug Information Services/ or Medication Therapy Management/ or Patient Care Team/ or ("pharmacist*" or "pharmaceutical intervention*" or "pharmaceutical care" or "pharmacies" or "pharmacist-led" or "team-based care").tw.  2. Hypertension/ or essential hypertension/ or hypertension, malignant/ or Antihypertensive Agents/ or [Blood Pressure](https://ovidsp.dc2.ovid.com/ovid-b/ovidweb.cgi?&S=NBMBFPELEGEBDCBLIPPJOEOGHANJAA00&Search+Link=%22Blood+Pressure%22%2f)/ or Blood Pressure Monitoring, Ambulatory/ or (hypertension or "high blood pressure" or "blood pressure management" or "blood pressure control" or "blood pressure monitoring" or "blood pressure telemonitoring" or "changes of blood pressure" or "hypertensive disease*" or "antihypertensive" or "antihypertensive agents" or "high bp" or "bp raised" or "bp control").tw. not ("pulmonary hypertension".tw.)  3. ((randomized controlled trial or controlled clinical trial).pt. or randomized.ab. or placebo.ab. or drug therapy.fs. or randomly.ab. or trial.ab. or groups.ab.) not ("trial registration number".tw.)  4. 1 AND 2 AND 3 |
| --- | --- |
| Embase Search Strategy | 1. 'pharmacist'/exp OR 'clinical pharmacy'/de OR 'evidence-based pharmacy'/de OR 'pharmacy research'/de OR 'medication therapy management'/de OR 'pharmaceutical care'/de OR 'pharmacist attitude'/de  2. 'hypertension'/de OR 'diabetic hypertension'/de OR 'essential hypertension'/de OR 'hereditary hypertension'/de OR 'hypertensive crisis'/de OR 'malignant hypertension'/de OR 'orthostatic hypertension'/de OR 'resistant hypertension'/de OR 'antihypertensive agent'/de OR 'elevated blood pressure'/de OR 'blood pressure monitoring'/de OR 'blood pressure measurement'/exp OR 'blood pressure regulation'/exp OR 'blood pressure fluctuation'/exp OR 'antihypertensive therapy'/de OR 'antihypertensive activity'/de OR 'hypertensive patient'/de  3. 'crossover procedure':de OR 'double-blind procedure':de OR 'randomized controlled trial':de OR 'single-blind procedure':de OR (random* OR factorial* OR crossover* OR cross NEXT/1 over* OR placebo* OR doubl* NEAR/1 blind* OR singl* NEAR/1 blind* OR assign* OR allocat* OR volunteer*):de,ab,ti NOT 'Conference Abstract'  4. NOT [medline]/lim  5. 1 and 2 and 3 not 4 |
| Cochrane Central Search Strategy | 1. Pharmacists or "Community Pharmacy Services" or "Pharmaceutical Services" or "Pharmaceutical Services, Online" or "Pharmacy Service, Hospital" or "Pharmacies" or "Pharmacy" or "Evidence-Based Pharmacy Practice" or "Pharmacy research" or "Drug Information Services" or "Medication Therapy Management" or "Patient Care Team" in Title Abstract Keyword  2. Hypertension or "essential hypertension" or "hypertension, malignant" or "Antihypertensive Agents" or "Blood Pressure Monitoring, Ambulatory" in Title Abstract Keyword - (Word variations have been searched) |
| JBI Search Strategy | 1. Pharmacist* or "Community Pharmacy" or "Pharmaceutical Services" or "Pharmacy Service" or Pharmacies or Pharmacy or "Evidence-Based Pharmacy Practice" or "Drug Information Services" or "Medication Therapy Management" or "Patient Care Team" or "Pharmaceutical intervention*" or "Pharmaceutical care" or "pharmacist-led" or "team-based care"  2. Hypertension or "Antihypertensive Agents" or "Blood Pressure Monitoring" or "high blood pressure" or "blood pressure management" or "blood pressure control" or "blood pressure telemonitoring" or "changes of blood pressure" or "hypertensive disease*" or "antihypertensive" |
| CINAHL Search Strategy | 1. (MH "Pharmacists") OR (MH "Pharmacist Attitudes") OR (MH "Pharmacy Service") OR (MH "Medication Management") OR (MH "Pharmacy, Retail") OR (MH "Drug Information Services") OR (MH "Prescription Drug Monitoring Programs") OR (MH "Medication Management") OR (MH "Multidisciplinary Care Team")  2. (MH "Hypertension") OR (MH "Essential Hypertension") OR (MH "Hypertension, Malignant") OR (MH "Hypertension, Isolated Systolic") OR (MH "Hypertensive Crisis") OR (MH "Antihypertensive Agents") OR OR (MH "Blood Pressure Determination") OR (MH "Blood Pressure Monitoring, Ambulatory")  3. (randomized controlled trials OR MH double-blind studies OR MH single-blind studies OR MH random assignment OR MH pretest-posttest design OR MH cluster sample OR TI (randomised OR randomized) OR AB (random*) OR TI (trial) OR (MH (sample size) AND AB (assigned OR allocated OR control)) OR MH (placebos) OR PT (randomized controlled trial) OR AB (control W5 group) OR MH (crossover design) OR MH (comparative studies) OR AB (cluster W3 RCT)) NOT ((MH animals+ OR MH animal studies OR TI animal model*) NOT MH human) |
| Web of science Search Strategy | 1. Pharmacist* or “Community Pharmacy” or “Pharmaceutical Services” or “Pharmacy Service” or Pharmacies or Pharmacy or “Evidence-Based Pharmacy Practice” or “Drug Information Services” or “Medication Therapy Management” or “Patient Care Team” or "Pharmaceutical intervention*" or "Pharmaceutical care" or "pharmacist-led" or "team-based care"  2. Hypertension or “Antihypertensive Agents” or “Blood Pressure Monitoring” or "high blood pressure" or "blood pressure management" or "blood pressure control" or "blood pressure telemonitoring" or "changes of blood pressure" or "hypertensive disease*" or "antihypertensive"  3. “randomized controlled trial*” OR “double-blind studie*” OR “single-blind studie*” OR “random assignment” OR “pretest-posttest design” OR “cluster sample” |
| Tripdatabase Search Strategy | pharmacist intervention hypertension  Filtre : rct |
| Grey literature Search Strategy | (pharmacist OR pharmacists) AND (hypertension OR antihypertensive OR "high blood pressure") |

**Supplemental Table S2.** Independently extracted data during the systematic review process.

| **Type of data** | **Items** |
| --- | --- |
| 1. Study identification | Author(s), year of publication, study country. |
| 2. Study characteristics | Setting and design.  Study duration, frequency of follow-up.  Randomization, blinding.  Sample size (total and per arm). |
| 3. Participants characteristics | Number of participants allocated to each group, number of patients analyzed.  Mean age, age range, sex.  Diabetes, other comorbidities, cardiovascular risk factors (smoking, dyslipidaemia).  Drug intake. |
| 4. Usual care (control group) characteristics | Healthcare providers involved.  Frequency of follow-up. |
| 5. Intervention characteristics | Type of interventions (pharmacist directed or in collaboration).  Duration of intervention.  Description of interventions: key components, frequency, format (noting if the detail provided is enough for replication), healthcare providers involved.  Cochrane Effective Practice and Organization of Care (EPOC) taxonomy. |
| 6. Outcomes for each group | Mean BP change and standard error (or confidence interval, p value) between baseline and follow-up.  Mean BP and SD at baseline and follow-up.  BP control (% reaching a pre-defined BP target) at baseline and follow-up.  Method of BP measurement. |

**Supplemental Table S3.** Study and pharmacist intervention characteristics for each included study.

| **Source; year of publication and country** | **Study setting** | **Study design, duration** | **Sample size (intervention/usual care) in analysis** | **Participants; mean age** | **Key components of pharmacist interventions** | **EPOC category (classified by VG, ST)** | **Intervention frequency, duration** | **Description of usual care group** | **Outcomes** |
| --- | --- | --- | --- | --- | --- | --- | --- | --- | --- |
| *Pharmacist directed care* | | | | | | | | | |
| McKenney et al; [1] 1973 United States | Community pharmacy | RCT, roughly 11 months | 45 (24/21) | Uncontrolled hypertensive patients (diastolic BP≥90 mmHg) taking antihypertensive Med; 60 y | Patient interview related to Med and prescriptions; Med education; DRPs identification and resolution; Distribution of additional educational material; Recommendation to physician regarding Med changes | Patient education  Healthcare provider feedback | Monthly, over 5 months | Throughout the study period, patients in both groups continued to receive medical care from one of the two health center physicians participating in the study as well as all other health services provided by Model Neighborhood  Comprehensive Health Program | Average SBP after the study period in the study group and control group=149 mmHg and 168 mmHg, respectively.  Average DBP after the study period in the study group and control group=97 mmHg and 103 mmHg, respectively.  A significant difference between groups was shown through a one-way analysis of variance on the differences in physician recorded BP in the control and study patients [F (1, 46) =21.988, p<0.001] |
| McKenney et al; [2] 1978 United States | Community pharmacy | RCT, 4 months | 136 (70/66) | Hypertensive patients seeing a physician for the treatment of their disease; 55 y* | Pharmacy training program (notebook on hypertension, tape recorded, self-assessment, on campus training, clinic visit, case study exam), monitoring and educational services during their regular visits to the pharmacy for refill of antihypertensive therapy (adverse reactions, compliance, inappropriate use, interactions, recording of BP) | Healthcare provider education  Healthcare provider feedback  Patient education | One training for pharmacists, patient intervention at every visit | Control patients received none of the services provided to the intervention group | Better BP control in study patients.  Control patients with DBP< 95 mmHg by the end of the study period=58%.  Study patients with DBP<95 mmHg by the end of the study period=74%.  Average DBP in the control group remained unchanged during the observation period. DBP in study patients dropped an average of 6 mmHg. A χ^2^ analysis of differences in the number of normotensive control and study patients showed a significant difference between groups (p<0.05) |
| Park et al; [3]1996  United States | Community pharmacy | RCT, 4 months | 53 (27/26) | Patients receiving antihypertensive Med or with BP≥ 140/90 mm Hg; 60 y | Drug therapy monitoring (BP recording, medication history, screening for drug interactions and adverse drug reactions, medication change recommendations and follow-up information to physician) and patient education (verbally and written compliance, lifestyle) | Patient education  Healthcare provider feedback | Monthly, 4 visits | Received traditional pharmacy services: screening for errors in prescribing, filling prescription orders, limited patient education, monitoring adverse drug reactions | SBP after the study period in the study group and in the control group=143.2 mmHg and 148.6 mmHg, respectively.  DBP after the study period in the study group and in the control group=83.2 mmHg and 83.7 mmHg, respectively |
| Carter et al; [4]1997 United States | Community pharmacy | RCT, 6 months | 55 (26/29) | Patients receiving antihypertensive Med or with a BP ≥ 140/90 mm Hg, 68 y | BP measurement; questions about ADRs, compliance, drug regimen, lifestyle; patient education (pamphlets, visual materials, verbal instructions - lifestyle, drug therapy); progress notes to physician | Patient education  Healthcare provider feedback | Visits every 3-5 weeks, over 6 months | Controls were seen only when they wanted prescription refills. Controls received traditional pharmacy services including brief counseling and prescription review for errors. | SBP after the study period in the study group and in the control group=140 mmHg and 143 mmHg, respectively.  DBP after the study period in the study group and in the control group=80 mmHg and 79 mmHg, respectively |
| Erickson et al; [5] 1997 United States | Outpatient clinic | RCT, 5 months | 80 (40/40) | Hypertensive patients (BP≥ 140/90 mm Hg) taking antihypertensive Med; 65 y | Medication review, patient education (compliance, drug therapy, lifestyle), consulting with physicians, BP measurement and interpretation | Patient education  Healthcare provider feedback | 5 months, regular visits | The control group received regular care without the interventions related to pharmaceutical care | Change in DBP=4.7mmHg and 2.6 mmHg for intervention and control groups, respectively (p=0.49).  Change in SBP= 12.0mmHg and 2.7 mmHg, respectively (p=0.05) |
| Solomon et al; [6] 1998 United States | Outpatient clinic, | RCT, 6 months | 133 (63/70) | Patients currently receiving dihydropyridine therapy or dihydropyridine and diuretic therapy for the treatment of hypertension; 18 years of age or older; signed the informed consent, could read and write in English; 67 y | Patient education (disease, drug therapy), collaboration with physician | Patient education  Healthcare provider feedback | 6-month treatment period, scheduled visits at enrollment and then at one-month intervals (four to six weeks) for a total of 5 visits | Control patients did not have access to the primary pharmacy caregivers and received no supplemental education or assessment of needs beyond what was customarily offered at each site. Traditional pharmacy care ranged from non-standardized interventions to distribution of product only | SBP after the study period in the study group and in the control group=138 mmHg and 144 mmHg, respectively (p=0.04).  DBP after the study period in the study group and in the control group=80.6 mmHg and 83.3 mmHg, respectively |
| Blenkinsopp et al; [7] 2000 United Kingdom | Community pharmacy | Cluster-RCT, 6 months | 282 (115/167) | Hypertensive patients taking antihypertensive Med; 50% of the patients were also treated for other conditions; 61 y* | Questioning protocol, patient education (verbal or written), contact or refer to GP | Patient education  Healthcare provider feedback | 3 visits, with 2-month interval | Usual care | Overall percentage of patients with uncontrolled BP prior to the study who had controlled BP after the study period=25.4%.  The percentage was higher in intervention patients (35.7%) than controls (17.1%) (p<0.05) |
| Mehos et al; [8] 2000 United States | Outpatient clinic | RCT, 6 months | 36 (18/18) | Uncontrolled hypertensive patients (BP 140-179/90-109 mmHg) taking at least one antihypertensive Med; 59 y | Education of HT and lifestyle using pamphlets; Distribution and education of home BP device with diary to document home BP values, change in HT Med and missed doses; Telephone contact to evaluate home BP measurements; Med recommendation to physician if BP ≥ 140/90 mmHg | Patient education  Patient reminder | Initial 30-minute appointment, contact every 1-2 months over 6 months | Control subjects were not given a home blood pressure monitor. They were allowed to continue monitoring at retail stores (pharmacy, grocery store) or at the medical clinic if they already were doing so. However, to maintain control conditions, they agreed not to start home monitoring during the 6 months of study. Primary care providers continued to make antihypertensive drug adjustments as part of routine care. Like the intervention group, controls were not restricted as to frequency of office visits with primary care providers | Between-group analysis of mean absolute decrease in SBP= 17.1 mmHg in intervention group and 7.0 mmHg in control group (p=0.069).  Between-group analysis of mean absolute decrease in DBP= 10.5 mmHg in intervention group and 3.8 mmHg in control group (p=0.022) |
| Okamoto et al; [9] 2001United States | Outpatient clinic | RCT, 6 months | 330 (164/166) | Controlled or uncontrolled hypertensive patients (mild to moderate HT) taking antihypertensive Med; 62 y | Counseling of Med; education of HT Med and non-Med; Verbal recommendations to physician regarding any Med changes | Patient education  Healthcare provider feedback | At pharmacists' or GPs' discretion | In the physician-managed clinic, physicians managed the treatment of patients in the control group independently with no pharmacy intervention | At final visit SBP= 135.10 mmHg in hypertension clinic and 141.66 mmHg in physician clinic (p=0.001).  At final visit DBP= 77.65 mmHg in hypertension clinic and 80.67 mmHg in physician clinic (p=0.004) |
| Garcao et al; [10] 2002 Portugal | Community pharmacy | RCT, 6 months | 82 (41/41) | Controlled or uncontrolled hypertensive patients taking antihypertensive Med; 65 y | Pharmaceutical Care Program including:  1. Monthly measure of BP;  2. HT and lifestyle habits education and counseling (educational leaflets about HT, food, and diet);  3. DRPs identification;  4. Recommendation to physician regarding Med regimen changes via letter or telephone | Patient education  Healthcare provider education | Monthly, over 6 months | Control patients received traditional pharmacy services consisting of brief counseling, medication review, and monitoring for adverse drug reactions. Control patients had their BP measured at baseline and after 6 months. The same method for BP measurement was used as for the intervention patients, and these patients contacted the pharmacist only when a medication refill was needed | Mean difference in SBP at the end of the study= -18.36 mmHg (p=0.0001)  Mean difference in DBP at the end of the study= -7.02 mmHg (p=0.001) |
| Vivian et al; [11] 2002 United States | Outpatient clinic | RCT, 6 months | 53 (26/27) | Uncontrolled hypertensive patients (BP≥140/90 mmHg) taking antihypertensive Med; 65 y | Counseling of Med and lifestyle; Assessment of compliance; Change in HT Med (drug selection and dosage) | Patient education  Healthcare provider feedback | Monthly | Patients in the control group received traditional pharmacy services (dispensing, brief counseling about drugs, and review of drug profiles) but did not make monthly visits to the pharmacist-managed hypertension clinic. They received care from their primary care providers as needed, at least once/year. All primary care providers were informed that their patients were enrolled in the study | SBP after the study period in the study group and in the control group=130.5 mmHg and 148.4 mmHg, respectively (p=0.0002).  DBP after the study period in the study group and in the control group=77.5 mmHg and 80.4 mmHg, respectively (p=0.259) |
| Murray et al; [12] 2004 United States | Outpatient clinic | RCT, 12 months | 351 (180/171) | Patients with a confirmed HT diagnosis or with BP≥140/90 and a prescription for at least 1 antihypertensive drug; 54 y | Pharmacist intervention recording system (PIRS software) | Healthcare provider reminder | Refill visits over 12 months | Usual care was provided by control pharmacists | SBP last 6-month measurements (study months 7-12) in the study group and in the control group=144 mmHg and 143 mmHg, respectively.  DBP last 6-month measurements (study months 7-12) in the study group and in the control group=77 mmHg and 78 mmHg, respectively |
| Sookaneknun et al; [13] Thailand 2004 | Community pharmacy | RCT, 6 months | 235 (118/117) | Controlled or uncontrolled hypertensive patients (BP≥140/90 mmHg or BP≥130/85 mmHg if patients had DM) taking antihypertensive Med or not; 63 y | Measurement of BP; Education and counseling of Med, disease, and lifestyle (educational leaflets and diary about HT and food); DRPs identification, resolution, and prevention; Recommendation to physician regarding Med regimen changes via letter or patient medical record | Patient education  Healthcare provider feedback | Monthly over 6 months | This group had no research pharmacist involvement. Control patients received the traditional service provided by the hospital or the primary care units. BP measurement was performed at the pre- and post-test periods. The same method of BP measurement was used as in the intervention group | Mean SBP at 6-month follow-up=121.47 mmHg in treatment group and 124.77 mmHg in control group, respectively (p=0.037).  Mean DBP at 6-month follow-up=71.55 mmHg in treatment group and 74.23 mmHg in control group, respectively (p=0.027) |
| Reid et al; [14] 2005 United Kingdom | Outpatient clinic | RCT, 5 months | 160 (92/68) | Hypertensive patients; 65y | Assessment of CHD/CVD risk, feedback to GP about medication changes, dose titration independently, patient education, clinical measurements (blood samples, ECG, urine samples) | Patient education  Healthcare provider feedback | 15 minutes appointments every 2 weeks to 3 months depending on BP control | Standard treatment through their GP for the initial 5 months and afterwards invited to the intervention (attend the Hypertension Management Clinic). Hypertensive patients not coded as such could also be referred directly to the clinic by their GP. | Number of patients meeting target level BP after 5 months: 74 (80.4%) in intervention group, 27 (39.7%) in control group, p<0.001 |
| Zillich et al; [15] 2005 United States | Community pharmacy | cluster- RCT, 3 months | 117 (57/60) | Uncontrolled hypertensive patients (BP 145- 179/95-109 mmHg; BP 135-179/90-109 mmHg if DM) taking 1-3 antihypertensive Med; 65 y | Trial of high-intensity intervention versus low-intensity intervention. High-intensity intervention: education of disease, HT Med, lifestyle modification and home monitoring of SBP; Handouts; Distribution of home BP device; Suggestion of drug adherence aids if necessary; Written treatment recommendations to physician regarding intensification of Med regimen; Contact with physician to develop treatment plan; Patient education related to treatment plan | Patient education  Healthcare provider feedback | 4 times over 3 months (at 4, 6-8 and 12 weeks) 15-60 minutes each | Patients at pharmacies met face-to-face with a trained pharmacist 3 times over 3 months. At each visit, patients’ BP was measured by the pharmacist. In most cases, patients were told that their BP was above normal, and they should contact their physician. These patients did not receive any other pharmacist education or home BP monitors. The BP measurements were sent via facsimile to the patients’ physician without treatment recommendations. There was no other contact with physicians | Difference in SBP change at the final visit between HI and LI groups=-4.5 mmHg (p=0.12)  Difference in DBP change at the final visit between HI and LI groups=-3.2 mmHg (p=0.03) |
| DeCastro et al; [16] 2006 Brazil | Outpatient clinic | RCT, 6 months | 71 (37/34) | Uncontrolled hypertensive adult patients (average of two office BP measurements ≥140/90 mm Hg) receiving treatment for HT at least with hydrochlorothiazide; 62 y | Pharmacist training, patient education (printed material), identification of drug-related problems, discussion with consultant physician in complex situations (Dader method), patient knowledge and use of drug and nondrug treatment | Healthcare provider education  Patient education | 5 meetings | Sham intervention (cognitive tests) | Adjusted δ-values between the intervention and control groups for ambulatory SBP in the different daily periods= 3 mmHg (95%CI=-1 to 5).  Adjusted δ-values between the intervention and control groups for ambulatory DBP in the different daily periods= 1 mmHg (95%CI=-1 to 3) |
| Hennessy et al; [17] 2006 United States | Outpatient clinic | Cluster-RCT, 12 months | 7159 (3617/354) | Controlled and uncontrolled hypertensive patients; 62 y | Academic detailing, provision of provider-specific data about hypertension control, provision of educational materials to the provider, and provision of educational and motivational materials to patients | Healthcare provider education  Patient education | 20-30 minutes visit, 2 mailings, 1 month apart | No intervention | Adjusted effect for SBP=-1.80 (95% CI= -3.97-0.37).  Adjusted effect for DBP=0.04 (95% CI=-1.05-1.12) |
| Green et al; [18] 2008 United States | Outpatient Clinic | RCT, 12 months | 519 (261/258) | Uncontrolled hypertensive patients (BP 140- 199/90-109 mmHg) taking antihypertensive Med; 59 y | Pharmacist care management using Web site including:  1. Home BP monitoring;  2. Patient education (instructions for home BP monitoring and Med);  3. Telephone call (Med history and cardiovascular risk factors);  4. Recommendation to physician regarding Med changes via Web communications (electronic copy to patients) | Healthcare provider education  Patient education | Every 2 weeks until control BP | After the first randomization, those assigned to usual care were told their BP was not in control and were encouraged to work with their physician to improve it | Adjusted mean change in SBP=-12.9 mmHg in pharmacist care and -5.1 mmHg in usual care (p=0.001).  Adjusted mean change in DBP=-6.2 mmHg in pharmacist care and -3.3 mmHg in usual care (p=0.001) |
| Planas et al; [19] 2009 United States | Community pharmacy | RCT, 9 months | 40 (25/15) | Patients with uncontrolled DM (HbA1c>7.0%) and HT (BP≥130/80mmHg) or taking antihypertensive Med; 65 y | A community-based Med therapy management program:  1. Med review related to current prescribed and non-prescribed Med to identify DRPs;  2. DRPs identification;  3. If DRP identified, recommendations to physicians regarding adjustment HTA Med dose and addition Med by fax or telephone;  4. Patient education regarding Med, lifestyle and diet;  5. A copy of the visit note sent to physician | Patient education  Healthcare provider feedback | Monthly | Participants in the control group attended visits at baseline and 3, 6, and 9 months, during which their BP was recorded, and they were informed of BP goals for patients with diabetes | Difference in SBP change between  the control and intervention groups= 20.05 mmHg (95%CI= 7.45 - 32.66) |
| Jamieson et al; [20] 2010 United Kingdom | Outpatient clinic | Cross-over trial 12 months | 33 (16/17) | Patients with a diagnosis of HT, with BP ≥140/85mmHg and receiving hypertensive Med, 59 y | Medication review, patient education (medication, conditions, lifestyle), pharmaceutical care plan with drug recommendation, monitoring tests | Patient education  Healthcare provider feedback | 6 visits (recruitment, baseline, 1, 2, 3, 6 months) | Routine management by their GP | SBP change over the treatment period in the study group and control group=12.5 mmHg mean decrease and 1.1 mmHg mean increase, respectively.  DBP change over the treatment period in the study group and control group=10.7 mmHg mean decrease and 2.3mmHg mean increase, respectively |
| Ashok Kumar et al; [21] 2011 India | Outpatient Clinic | RCT, 8 months | 100 (50/50) | Hypertensive patients aged between 18 and 65 years** | Patient education (hypertension knowledge assessment questionnaire, pamphlet, pictures) | Patient education | Once | No counseling | After four months of intervention 63.64% (n=22) and 64.2% (n=28) experimental men and women achieved normal levels of BP, respectively compared to 28.58% (n=28) and 40.91% (n=22) control men and women, respectively |
| Magid et al; [22] 2011 United States | Outpatient clinic | RCT, 6 months | 338 (174/164) | Uncontrolled hypertensive patients (BP>140/90 mmHg or BP>130/80 mmHg if DM or CKD) taking 4 or fewer antihypertensive Med; 66 y | Patient education, home BP monitoring, home BP measurement reporting to an interactive voice response phone system, clinical pharmacist management of hypertension with physician oversight | Patient education  Healthcare provider feedback | Weekly BP report and review | Usual care (*no further information provided*) | Difference in SBP for intervention patients compared with usual care patients= −6.0 (95% CI= −10.4 to −1.7) mm Hg.  Difference in DBP for intervention patients compared with usual care patients= −2.3 (95% CI= −4.9 to −0.2) mm Hg |
| Morgado et al; [23] 2011 Portugal | Outpatient clinic | RCT, 9 months | 197 (98/99) | Controlled or uncontrolled hypertensive patients taking antihypertensive Med for at least 6 months; 60 y | Pharmacist intervention program including:  1. Patient interview identifying problems leading to poor BP control;  2. Patient education (with written information) and counseling regarding hypertension, lifestyle, and compliance (with propositions of tips to enhance compliance);  3. Assessment of Med compliance by returning empty blisters and boxes of antihypertensive Med;  4. Recommendation to physician regarding Med changes | Patient education  Healthcare provider feedback | At baseline,3, and 6 months | The control group had no clinical pharmacist involvement and control patients received the traditional service provided by the hospital clinic | ITT SBP=134.2 mmHg in the intervention group and 141.0 mmHg in the control group (p=0.006).  ITT DBP=82.5 mmHg in intervention group and 85.4 mmHg in control group (p=0.020) |
| Skowron et al; [24] 2011 Poland | Community pharmacy | Cluster-RCT, 14 months | 84 (56/28) | Hypertensive adult patients pharmacologically treated for at least 6 months; 60 y* | Pharmacist training (DRPs, hypertension pathophysiology, lifestyle, pharmacotherapy), BP measurement, patient counseling (DRPs, patient education) | Patient education  Healthcare provider feedback  Healthcare provider education | 12 meetings, over 14 months | Pharmacists from the control group did not do any additional services for patients included into the study, so they did not monitor the pharmacotherapy and did not educate the patients | SBP after the study period in the study group and in the control group=138 mmHg and 142 mmHg, respectively (p=0.5).  DBP after the study period in the study group and in the control group=83 mmHg and 88 mmHg, respectively (p=0.4) |
| Wang et al; [25] 2011 China | Outpatient clinic | RCT, 12 months | 59 (29/30) | Uncontrolled hypertensive patients (BP≥140/90 mmHg) for at least 1 year and taking antihypertensive Med; 48 y | Pharmaceutical care interventions including patient education on Med (indication, side-effects, and usage instructions), compliance and healthy lifestyle behaviors (e.g., vegetarian diet, sodium intake, weight, and physical activity); Identification of DRPs and communication with physicians to resolve DRPs, if necessary | Patient education  Healthcare provider feedback | Every 2  months | The participants returned to the hospital every 2 weeks for regular clinical follow-up, including the evaluation of BP. Nursing staff followed the participants by telephone once a month and recorded the participants’ blood pressure and medication compliance | Change in 24-h SBP= -9.75 mmHg in the intervention group and 1.07 mmHg in the control group (p=0.036)  Change in 24-h DBP= 5.88 mmHg in intervention group and 1.67 mmHg in control group (p=0.040) |
| Heisler et al; [26] 2012 United States | Outpatient clinic | Cluster-RCT, 26 months | 4100 (1797/2303) | Patients with DM and HT with poor BP control; 65 y | Pharmacist training (motivational interviewing training), medication changes, patient counseling (BP self-monitoring, adherence barriers, discussed lab values), BP measurement | Healthcare provider education  Patient education | Scheduled visits over 14 months until discharge (all medication adherence issues had been addressed; home or clinic BPs were at target; or the patient was on maximum tolerated medications) | Standard healthcare services through their primary care provider, which in all sites included access to care manager and other control clinical pharmacist services targeting patients with diabetes mellitus with poor risk factor control. The study team had no contact with the usual care teams, nor did the intervention clinical pharmacists who worked exclusively with intervention team patients | In the primary analysis, the intervention group SBP change from the 6 months before versus 6 months after the 14-month intervention was not different from control group, declining 8.9 mm Hg in the intervention group in comparison with a 9.0 mm Hg decrease for the control group (difference of 0.18 [0.77, 1.13]) |
| Ramanath et al; [27] 2012 India | Outpatient clinic | RCT, 7 months | 52 (26/26) | Adult hypertensive patients on Med for HT over a period of 6 months; 57 y* | The intervention group patients were counselled on various aspects such as, drugs, lifestyle changes, and their disease management, and told them to inform if any unwanted and unintended effects of drugs occur at any follow-ups. | Patient education | Monthly, 3 visits | The control group did not provide with any counselling and patient information leaf lets at the baseline and in the first follow‑up | SBP at second follow-up=128.27 mmHg in intervention group and 131.08 mmHg in the control group (p= 0.086).  DBP at second follow-up=77.73 mmHg in intervention group and 78.46 mmHg in control group (p=0.5) |
| Zaragoza-Fernandez et al; [28] 2012 Spain | Community pharmacy | RCT, 2 months | 143 (71/72) | Hypertensive, treatment-compliant patients who are not controlled with antihypertensive agents; 68 y | Patient education with handout (diet, salt intake, alcohol intake and exercise); phone calls to patients; personal interview (lifestyle changes, problems) | Patient education  Patient reminder | Weekly phone call over 3 weeks, 1 personal interview in week 4, weekly phone calls over 3 weeks, 1 personal interview in week 8 | Routine treatment | Mean SBP at week 8=131.6 mmHg in the intervention group and 142.0 mmHg in the control group (p<0.001).  Mean DBP at week 8=81.4 mmHg in the intervention group and 87.1 mmHg in the control group (p<0.001) |
| Zhao et al; [29] 2012 China | Outpatient clinic | RCT, 6 months | 258 (129/129) | Controlled or uncontrolled hypertensive patients taking antihypertensive Med for at least 6 months; 64 y | Hypertension staging, risk stratification; determination of a goal BP and treatment recommendations to the patient’s physician; educating patients and family members; monitoring adverse drug reactions and adherence | Patient education  Healthcare provider feedback | Scheduled visits every 2 months over 6-month period, plus optional visits | The control group had no clinical pharmacist involvement and patients received traditional service provided by the hospital clinic | Mean SBP at the end of the study period=134.0 mmHg in the intervention group and 142.1 mmHg in the control group (p=0.0001).  Mean DBP at the end of study period= 80.5 mmHg in the intervention group and 84.6 mmHg in the control group (p =0.0013) |
| Magid et al; [30] 2013 United States | Outpatient clinic | RCT, 6 months | 348 (175/173) | Uncontrolled hypertensive patients (BP≥140/90 mmHg or BP≥130/80 mmHg if DM or CKD) taking ≤3 antihypertensive Med); 60 y | After receiving instructions on home BP device, pharmacist intervention included:  1. Meeting with patients to review antihypertensive Med and to counsel on lifestyle changes;  2. Reviewing of home BP measurements and adherence to Med;  3. Adjustments or changes of antihypertensive Med as needed;  4. Recommendation to physician regarding Med changes via electronic health record;  5. Communication with patients via telephone or secure e-mail | Healthcare provider feedback  Patient reminder | Every patient encounter | Patients assigned to the usual care group were advised that their BP was elevated; received written educational materials on managing high BP, diet, and physical activity; and were instructed to follow up with their primary care physician. In addition, the patient's physician was notified of the patient's elevated BP via a note sent to the electronic health record in-box of the physicians | SBP after 6 months=128.1 mmHg in the HBPM group and 137,4 mmHg in the UC group (p<0.001).  DBP after 6 months=79.1 mmHg in the HBPM group and 83,1 mmHg in the UC group (p<0.01) |
| Margolis et al; [31] 2013 United States | Outpatient clinic | Cluster-RCT, 12 months | 370 (188/182) | Patients with uncontrolled HT (BP≥140/90 mmHg) at the most recent primary care visits in the previous year taking antihypertensive Med; 61 y | Face-to-face interview with patient including:  1. Patient education on using the home BP telemonitoring system, disease (hypertension)  and reviewing of patient's Med history;  2. Telephone contact with patient every 2 weeks until BP control was sustained with patient education on lifestyle change and Med adherence;  3. Assessment and adjustments of Med based on an algorithm;  4. Recommendation to physician via electronic health record at each visit | Patient education  Patient reminder | Every 2 months, then monthly | During the study period, usual care patients worked with their primary care physicians as they had in the past. This could include referral to a medication therapy management pharmacist for consultation (1-2 visits without telephone follow-up or prolonged monitoring) and conventional home BP measurement | Differential change from baseline at 18 months in SBP= -6.6 mmHg (-10.7 to -2.5).  Differential change from baseline at 18 months in DBP= -3.0 mmHg (-6.3 to 0.3) |
| Ramanath et al; [32] 2013 India | Outpatient clinic | RCT, 9 months | 90 (45/45) | Adult hypertensive patients with HT alone or up to 3 other comorbidities (DM, asthma, and COPD), 61 y* | Intervention group patients were counselled (verbal and nonverbal/written) on various aspects like disease, drugs, lifestyle modifications (e.g., low salt intake, exercise/walking, etc.) and their management during all the three follow-ups | Patient education | Monthly, 4 meetings | Control group patients were not counselled at the baseline but their knowledge, attitude and practices score, brief medication questionnaire and quality of life were assessed using their respective questionnaires. The answers given by them were recorded and the same method was carried out in first, second, and third follow-up. The control group was counselled at the third follow-up | SBP at third follow-up=130.89 mmHg in intervention group and 127.69 mmHg in control group (p= 0.021)  DBP at third follow-up=81.38 mmHg in the intervention group and 81.07 mmHg in the control group (p 0.604) |
| Wal et al; [33] 2013 India | Outpatient clinic | RCT, 6 months | 102 (54/48) | Hypertensive patients aged 20 to 75 years with an average DBP > 90 mmHg or an average SBP > 140 mmHg with or without other co‑morbidities; 60 y | Written health education material, patient counselling (medication, lifestyle, ADRs), BP measurement | Patient education | 3 visits, every 3 months | The control group did not receive any pharmaceutical care, and only the patient details were taken from the medical records and entered the patient data collection form | Student t‑test for independent samples showed a  significant difference of 12.24 mmHg (p=0.0001) between variation of means of SBP in the intervention group at the end of the study and a significant difference of 5.17 mmHg in DBP (p=0.001) |
| Wong et al; [34] 2013 China | Outpatient clinic | RCT, 6 months | 231 (92/139) | Patients aged 18 years or older taking at least one long‐term antihypertensive agent with poor Med adherence; 62 y | Participants received both usual-care followed by community‐based medication counselling service immediately after physician consultation. The service is delivered by community pharmacists from a Non‐Governmental Organization. Patient education about disease and medication; provision of medication knives and pill boxes; educational pamphlets; offering of free‐of‐charge telephone consultations with community pharmacists | Patient education | 1 time; Sessions lasted 15–20 minutes | Educated on their diagnoses of hypertension, their implications, and discussed on the importance of proper medication compliance to control hypertension by the attending physicians. Most of these sessions lasted for 2–3 minutes | SBP at 6 months =131 mmHg in intervention group, and 131 mmHg in usual care (p=0.947)  DBP at 6 months= 76.8 mmHg in intervention group and 78.2 mmHg in usual care (p=0.238) |
| DiDonato et al; [35] 2014 United States | Community pharmacy | RCT, 4 months | 180 (86/94) | Patients of 18 years of age or older with a diagnosis of HT inferred by Med use, with at least one prescribed BP Med, and who filled two or more prescriptions at the study site; 66 y | Appointment based model (monthly medication review, reminder, BP measurement); Education group (monthly education session) | Patient reminder  Patient education | Monthly, 4 visits | Control group patients did not receive additional intervention beyond monthly BP checks assessed on a walk-in basis. They were not reminded to come to the pharmacy to refill medications or to have their BP measured | SBP, month 4= 128 mmHg in control group, 133 mmHg in the medication synchronization group and 129 mmHg in the education group.  DBP, month 4= 76 mmHg in the control group, 77 mmHg in the medication synchronization group and 76 mmHg in the education group |
| Stewart et al; [36] 2014 Australia | Community pharmacy | Cluster-RCT, 6 months | 352 (176/176) | Adults aged over 18 years with primary HT who have used antihypertensives in the previous 6 months; 67 y | Home BP monitor; Training by the pharmacist on self-monitoring of BP; Motivational interviewing and education by the pharmacist to help patients improve their medication adherence and achieve target BP; Pharmacy-based medicines review to identify and resolve, where necessary, possible medication-induced hypertension; Pharmacist-initiated dose administration aid, home-based medicines review and/or patient medication list, where necessary; Referral to a GP at the pharmacist’s discretion; Refill reminders (by SMS, telephone, or mail), if they so choose, from their pharmacist 3 days before their antihypertensive medication was due to run out | Patient education  Patient reminder | Baseline, at 3 months, at 6 months | Routine care (*no further information provided*) | Difference between usual care and intervention group in SBP change between baseline and 6 months= -5.3 mmHg (p=0.05).  Difference between usual care and intervention group in DBP change between baseline and 6 months=-0.3 mmHg (p=0.85) |
| Firmino et al; [37] 2015 Brazil | Outpatient clinic | RCT, 9 months | 68 (34/34) | Patients diagnosed with HT who had a) uncontrolled BP and/or presence of cardiovascular risk factors, or b) adherence problems to the treatment; or c) the need for education about the pharmacotherapy; 60 y | Pharmacotherapeutic follow-up performed by a pharmacist trained in pharmaceutical care practice, in addition to traditional care performed in the pharmacy unit. During the referred pharmacotherapeutic follow-up, activities in health education, detection, resolution, and prevention of DRPs, and monitoring of adherence to prescribed treatment were provided to the participants | Patient education  Healthcare provider feedback | 6 visits, every 1-2 months | The control group was only monitored by the researcher during the nine months aiming to collect data for future comparison with the intervention group | BP control at baseline= 60% in usual care and 46.2% in the intervention group.  BP control at follow-up= 56.7% in usual care and 65.4% in the intervention group |
| Hedegaard et al; [38] 2015 Denmark | Outpatient clinic | RCT, 6 months | 516 (231/285) | Patients aged 18 years or older who were prescribed at least one antihypertensive agent; 61 y | Medication review focused on identifying DRPs for antihypertensive or lipid-lowering agents followed by advice to the physician in charge; a patient interview; 2 or more follow-up telephone calls to the patient within the first 6 months after inclusion | Patient education  Healthcare provider feedback | 3 interviews (baseline, 1, 6 months), additional telephone calls if necessary | Both groups received usual care, which included 2-4 outpatient consultations with physicians or nurses per year. At the consultations, a broad range of risk factors, including lifestyle and adherence, were addressed. BP, blood glucose, and lipid profiles were measured, and adjustments of the medications were made. Clinical pharmacists were not involved in usual care | Change in SBP (95% CI)= -1.1 (-5.0-2.8) mmHg  Change in DBP (95% CI)= 0.4 (-2.2-3.1) mmHg |
| Saleem et al; [39] 2015 Pakistan | Outpatient clinic | RCT, 10 months | 264 (120/144) | Patients aged 18 or over with HT, and on antihypertensive Med for the last 6 months; 39 y | At each visit, the hospital pharmacist conducted a thorough interview with the patient, identified problems leading to poor medication adherence and provided patient education. Patients in the intervention group were also provided with a pocket-sized educational book on hypertension, information leaflets and medication adherence cards during the counselling process | Healthcare provider education  Patient education | Twice per month, over 9 months, first visit 15 minutes, follow-up visits 10 minutes | The control group had no hospital pharmacist involvement, and control patients received the traditional service provided by the hospitals (receiving prescription orders, counselling about medication use and information about follow-up visits) | Follow-up SBP in intervention group 137.5 mmHg, in control group 143.9 mmHg (p=0.004)  Follow-up DBP in intervention group 84.6 mmHg, in control group 90.1 mmHg (p=0.009) |
| Tsuyuki 2015 et al; [40] Canada | Community pharmacy | RCT, 6 months | 248 (181/67) | Adult outpatients with uncontrolled BP (BP≥140/90 mmHg or BP≥130/80 mmHg if DM); 64 y | The intervention group received enhanced pharmacist care, which was guided by the Canadian Hypertension Education Program guidelines and consisted of pharmacist assessment of and counselling about cardiovascular risk and BP control, review of antihypertensive medications, and prescribing/titrating of drug therapy if deemed necessary, in addition to a wallet card for recording BP measurements, lifestyle advice, and written information on hypertension. The patient’s primary care physician was notified of all assessment results and drug therapy changes in person or by fax. | Patient education  Healthcare provider feedback | Monthly intervals until BP was at target for 2 consecutive visits and thereafter at 3-month intervals for the duration of the study period (6 months) | The usual care group received a wallet card for recording BP, lifestyle advice as required, written information on cardiovascular disease, and BP measurement by the pharmacist at 3-month intervals. Patient education was provided at the discretion of each pharmacist. Patients’ primary care physicians were sent a notice that the patients were enrolled in the study based on their elevated BP, and patients were advised to see their physicians for further treatment. All patients were followed up for a total of 6 months | Adjusted difference in SBP over 6 months between intervention and usual care=6.6 mmHg (SE=1.9)  Adjusted difference in DBP over 6 months between intervention and usual care=3.2 mmHg (SE=1.3) |
| Bajorek et al; [41] 2016 Australia | Community pharmacy | Cluster RCT, 12 months | 34 (18/16) | Patients aged ≥18 years with non-controlled essential HT; 67 y | BP measurement, medication management review, medicine review, adherence support, recommendations to GP | Patient education  Healthcare provider feedback | 4 visits (baseline, 1, 3, 12 months), patient follow-ups via telephone (2 weeks, 6 months, 9 months) | Patients received usual care upon presentation to the pharmacist for refill prescriptions, i.e., supply of their medication and any medication counselling | SBP at 12 months= 125 mmHg in the control group and 132 mmHg in the intervention group.  DBP at 12 months= 75 mmHg in the control group and 73 mmHg in the intervention group |
| Kandasamy et al; [42] 2016 India | Outpatient clinic | RCT, 6 months | 60 (30/30) | Patients diagnosed with HT of either sex, aged between 20 and 80 years with or without comorbidities; 58 y* | Patient education (audiovisual session about hypertension, lifestyle), drug compliance | Patient education | Monthly, 3 visits | Usual care | SBP at the end of study period=145.33 mmHg in the intervention group and 150.50 mmHg in the control group.  DBP at the end of study period=86.67 mmHg in the intervention group and 87.77 mmHg in the control group |
| Lam et al; [43] 2017 United States | Community pharmacy | RCT, 3 months | 134 (68/66) | Adult patients with HT filling prescriptions to the study coordinator on site.; 70 y | Talking Pill Bottle | Patient reminder  Patient education | Monthly (baseline, 30, 60, 90 days) | Counselling to all study participants at point of dispensing based on the American Society of Health-System Pharmacists guidelines, regardless of standard treatment or intervention arm assignment or whether the prescription was for a new or refill medication | In the between-group analysis, the BP readings at 90 days between the standard treatment arm (SBP=139.63, SD=15.29; DBP=75.23, SD=10.52) and the intervention arm (SBP=138.89, SD=19.37; DBP=74.71, SD=13.00) were not significantly different (SBP: p=0.810;  DBP: p=0.801) |
| Amer et al; [44] 2018 Pakistan | Outpatient Clinic | RCT, 3 months | 384 (192/192) | Patients aged 30 years or older diagnosed with HT taking treatment for HT for the last six months; 50 y* | Pharmacist identified those issues leading to the poor adherence towards medication and provided disease related education to the patient (hypertension-related information, lifestyle education, medication counselling tips to increase knowledge about hypertension, adherence to medication and health related quality of life). A printed booklet (in Urdu language) of hypertension related educational material was provided to the patients in the intervention group | Patient education | 3 times, every 1.5 months, 15 minutes baseline, 10 minutes follow-up | No educational sessions were provided to the control group during the study duration. The control group received standard care only (provided by physician during scheduled visit to hospital) | SBP at the end of study period=131.81 mmHg in the intervention group and 137.91 mmHg in the control group (p<0.001).  DBP at the end of study period= 83.75 mmHg in the intervention group and 87.77 mmHg in the control group (p <0.001) |
| Cheema et al; [45] 2018 United Kingdom | Community pharmacy | RCT, 6 months | 64 (31/33) | Participants over 18 years on BP Med; 62 y | Individually tailored information sheets containing structured advice on BP and their anti-hypertensive medication that was prepared using National Institute for Health and Care Excellence guidance CG 127 | Patient education | Visits at 0,2,4,26 weeks | The participants in the control group received a separate information sheet containing information on the New Medicines Service in addition to usual care. | Effect on systolic BP was not sustained at the 26-week follow-up with little difference remaining between the groups, 8 mmHg (95% CI= 2.1–13.3, p=0.01) intervention group vs. 7 mmHg (95% CI= 0.6–11.7, p=0.02) control group.  For diastolic BP, there was no added effect of the intervention, and both groups achieved a similar reduction in diastolic BP at the 26-week follow-up, 4.5 mmHg (95% CI=1.2–7.7, p=0.008) intervention group vs. 5 mmHg (95% CI=1.3–8.8, p=0.009) |
| Okada et al; [46] 2018 Japan | Community pharmacy | Cluster RCT, 3 months | 125 (64/61) | Hypertensive patients aged 20-75 years who visited pharmacies to obtain Med for more than three months; 64 y | Pharmacist training (BP measurement, motivational advice), patient training and registration (sodium intake, more vegetables, exercising, losing weight, reducing alcohol) | Healthcare provider education  Patient education | 5 visits, over 12 weeks | Patients in the control group were provided the same home BP monitor as the Intervention group and a basic explanation about their medication. The attending pharmacists checked and evaluated their BP and measurement methods without providing special consultations | Mixed-effect model for repeated measures for morning SBP estimated difference= -4.5 mmHg (-8.5 to -0.6).  Mixed-effect model for repeated measures for morning DBP estimated difference= -1.8 mmHg (-4.4 to 0.8) |
| van der Laan et al; [47] 2018 Netherlands | Community pharmacy | RCT, 9 months | 170 (85/85) | Patients aged 45–75 years using antihypertensive Med and considered non-adherent based on both pharmacy dispensing data and a self-report questionnaire; 61 y | Tailored intervention guide (providing information, providing tools, dealing with side effects, overcoming problems, diminishing negative beliefs) | Patient education  Healthcare provider feedback | 2 consultations 3 months in between | Usual care according to the Dutch guidelines of the Royal Dutch Pharmacists Association. This care, usually delivered by a pharmacy technician, consist of checking and dispensing of prescribed drugs, providing instructions on medication use, and providing information about intended effects and possible side effects, during first and second dispensing. BP measurements in the control group were executed by the pharmacy technician | Adjusted SBP difference=1.52 mmHg (-2.71 to 5.75)  Adjusted DBP difference=0.63 mmHg (-2.65 to 3.92) |
| Goruntla et al; [48] 2019 India | Outpatient clinic | RCT, 6 months | 192 (95/97) | Patients aged ≥ 18 years, suffering from HT with or without comorbidities; 44 y | The counselling focused on hypertension definition, regular monitoring of BP and body weight, Dietary approach to stop hypertension diet, physical exercise, stress management, salt restriction, lifestyle changes (Smoking and alcohol), and regular intake of medications as per physician instructions. | Patient education | 3 visits (baseline, 3, 6 months) | The participants in the control group will follow the usual care given by the physician | SBP at final follow-up visit= 138 mmHg in the intervention group and 146 mmHg in the control group (p=0.039).  DBP at final follow-up visit= 82.3 mmHg in the intervention group and 87.2 mmHg in the control group (p=0.047) |
| Alfian et al; [49] 2020 Indonesia | Outpatient Clinic | Cluster RCT, 3 months | 113 (56/57) | Patients with DM2 and HT aged at least 18 years old, diagnosed with DM2 for at least 1 year, using at least one antihypertensive drug in the last 3 months and non-adherent to the HT treatment; 63 y* | Discussed patient-specific barriers for medication adherence. Simple question-based flowcharts and adherence intervention wheel provided to pharmacy staff. Goal setting. | Patient education | 2 visits (baseline, 1 months) | Received pharmacist counselling based on the Indonesian guideline of pharmacy practice | Mean difference in SBP=5.98 mmHg, 95%CI= -10.8, 22.76.  Mean difference in DBP= -8.61 mmHg, 95%CI= -20.01, 2.78 |
| Manigault et al; [50] 2020 United States | Outpatient clinic | RCT, 3 months | 55 (39/16) | Patients with at least 18 years of age, diagnosed with HT, prescribed at least one antihypertensive Med for a minimum of 3 months prior to enrolment; 44 y* | Customized mobile app (BP-n-Me): calendar reminders of medications, "call your pharmacy" button, BP log, lifestyle survey, patient education | Patient reminder  Patient education | 3 months | The limited version of the BP-n-Me app only allowed participants to enter their BP values and did not provide any additional functionality or app engagement | Median difference between groups in SBP= -2.00 (p=0.07)  Median difference between groups in DBP= -1.00 (p=0.39) |
| Zhai et al; [51] 2020 China | Outpatient clinic | Cluster RCT, 3 months | 384 (192/192) | Adults aged 18 years or over with HT, using antihypertensive Med; 69 y | Personal consultations by trained pharmacy students that lasted about 5 min to identify the reasons for medication nonadherence; SMS text messages sent at 3-day intervals | Patient reminder  Patient education | SMS at 3-day intervals over 3 months | Patients in the control group received a welcome SMS text message and an end-of-trial SMS text message, but they did not receive a personal consultation. Besides, both patients in the intervention group and control group were given standard pharmaceutical care according to the Guidelines for Good Pharmacy Practice | Difference in mean change between baseline and 3 months for SBP= -6.86 (95%CI= -10.37 to -3.34)  Difference in mean change between baseline and 3 months for DBP= -0.95 (95%CI= -1.09 to 2.98) |
| Jackson et al; [52] 2021 Nigeria | Outpatient clinic | RCT, 12 months | 206 (103/103) | Patients from 18 to 69 years old, diagnosed of HIV infection and HT, who had been on antiretroviral and antihypertensive drugs for at least three months prior to the start of the study; 48 y | Pharmaceutical care involving structured education and counselling by the research pharmacist after seeing the physician on their clinic visits. Patient education/counselling focused on self-monitoring of BP, healthy eating, maintaining an ideal body weight, proper foot and dental care, healthy coping, use and storage of medications; lifestyle modifications with emphasis on the dietary approaches to stop hypertension diet, physical activity, smoking cessation, moderation of alcohol intake; reviewing date of the next appointment and prescription with the patient, expected side effects of the medications, and how to prevent/manage such side effects. | Patient education  Healthcare provider feedback | bimonthly face-to-face appointments, text messages twice a month, over 12 months | Participants in the control arm received routine care: On their clinic appointment visits, the nurses take their BP measurements before they are seen by the physician who prescribes appropriately and if appropriate, orders relevant laboratory test(s). The monitoring and evaluation unit then gives a date for the next clinic appointment based on the duration of antiretroviral drugs prescribed. The patient sees the adherence counsellor who counsels or commend the patient based on their recent viral load or CD4+ cell count. Finally, the pharmacist dispenses prescribed medications and counsels the patient appropriately | ITT analysis for SBP= 137.6 mmHg in intervention group and 150.9 mmHg, in control group (p=0.001).  ITT analysis for DBP= 84.4 mmHg in intervention group and 90.5 mmHg in control group (p=0.001) |
| Khiali et al; [53] 2021 Iran | Outpatient clinic | RCT, 6 months | 122 (61/61) | Patients aged between 18 and 80 years with uncontrolled BP (BPs≥140 and/or 90 mmHg); 51 y | Patient education in the clinic (BP measurement), patient education and monitoring in their home (BP measurement, new medication) | Patient education  Patient reminder | weekly phone calls, daily BP measurement | In the usual care group, two attending cardiologists visited the patients twice weekly at the clinic. The conventional office-based BP control method was used as the control group in our study | Mean difference between baseline and 6 months after study of SBP= -34.9 mmHg in intervention group and -33.6 mmHg in control group (p=0.63).  Mean difference between baseline and 6 months after study of DBP= -18.9 mmHg in intervention group and -16.3 mmHg in control group (p=0.41) |
| Li et al; [54] 2021 China | Community pharmacy | RCT, 6 months | 588 (290/298) | Patients aged 18 years or older with a confirmed diagnosis of HT taking antihypertensive Med or having coexisting chronic conditions or reporting confusion with their own Med regimen or missing medicines frequently; 66 y | Medication review, medication adjustment plan, appointments (outpatient appointments, home visits, telephone interviews), medication recommendation to patient and GP, individual education plan | Patient education  Healthcare provider feedback | monthly medication adjustment plan, maximum 5 phone interviews each month, for 3 months, 6-10 minutes per interview | All the participants received the usual care and continued their community systematic management program delivered by a team comprising GPs, nurses, and public health workers | SBP at sixth month= 139.29 mmHg in intervention group and 143.54 mmHg in control group (p= 0.001).  DBP at sixth month= 77.94 mmHg in intervention group and 85.47 mmHg in control group, (p=0.001) |
| Liu et al; [55] 2021 China | Community pharmacy | RCT, 12 months | 196 (93/103) | Patients aged 18 year or more with HT; 69 y* | BP measurement, drug cards (daily self-recording), monthly home visits, medication review, patient education | Patient reminder  Patient education | Monthly visits, over 12 months | The non-intervention group received only routine medical services, including issuance of drugs and drug consultations | Percentage of participants with normal blood pressure status at first visit 8.7% in intervention group, 31.2% in non-intervention group  Percentage of participants with normal blood pressure status at last visit 43.7% in intervention group, 20.4% in non-intervention group |
| Mehas et al; [56] 2021 United States | Community pharmacy | RCT, 6 months | 28 (13/15) | Patients aged between 18 to 85 years with BP of >140/90 mm Hg (>130/80 mm Hg for patients with DM or CKD), receiving at least 1 antihypertensive Med with no change in the regimen or dose in the past 4 weeks, and receiving antihypertensive Med regularly from the pharmacy during the past 6 months; 60 y | Electronic reminder cap system named SMARxT cap (alert with beeps and flashes, visual timer, recorder) | Patient reminder | Over 6 months | The matching placebo cap physically resembles the SMARxT cap and records bottle openings but does not exhibit the audio or visual alerts | From baseline to 6 months SBP decreased by 8.2 mm Hg in the SMARxT cap group and 2.8 mm Hg in the placebo cap group (p=0.35).  For DBP, a decrease of 6.2 mm Hg was observed in the SMARxT cap group versus no change in the placebo cap group (p=0.06) |
| Contreras-Vergara et al; [57] 2022 Mexico | Outpatient clinic | RCT, 6 months | 89 (46/43) | Patients between 18 and 60 years old with HT and DM; 56 y | Patient education (disease, lifestyle, medication, orally and written, wallet card) | Patient education | 3 visits (baseline, 3, 6 months), for 20-25 minutes | Control group received regular (standard) education from their physician at each of their office visit | SBP at the end of study period=141.14 mmHg in control group and 130.15 mmHg in the intervention group.  DBP at the end of study period=92.91 mmHg in control group and 87 mmHg in the intervention group |
| Gupta et al; [58] 2022 United States | Outpatient clinic | RCT, 3 months | 31 (17/14) | Patients aged ≥65 years with uncontrolled HT (SBP >140 mmHg at visit and documented history of HT; SBP >140 mmHg at visit and at another visit in the last 18 months, or SBP >160 mmHg at visit), including men and women, all racial and ethnic minorities, patients with multiple comorbidities, frailty, mobility limitations; 71 y | Virtual collaborative care clinic: patient education (home BP monitoring, adherence, medication), communication with physician, order laboratory tests, e-prescribe medications, make medication changes | Patient education | At least once per months communication between patient and pharmacist, over 3 months | Usual care with education, continued regular follow-up with their physician | Patients in the intervention arm had a decrease in average SBP from baseline to 12-week visit (p=0.01) but patients in usual care arm did not (p=0.45) |
| Malik et al; [59] 2022 Pakistan | Community pharmacy | Cluster RCT, 6 months | 80 (40/40) | Patients diagnosed with DM (Type I or II) and HT with an HbA1c value ≥ 7% and BP greater than 140/90 mmHg at the time of diagnosis; 44 y* | Training aids for pharmacists (diabetes, hypertension, log sheets, glucometer, BP measuring devices, questionnaires), patient kits (disease brochures, diet charts, BP and glucose monitoring cards), oral patient counselling, BP and blood glucose measurement | Healthcare provider education  Patient education | Every 15 days, for 6 months, minimum 20 minutes | Control group received the usual pharmacy services, i.e., dispensing medications and providing information regarding medication administration. Patients enrolled in the control and intervention group were required to visit the community pharmacy every 15 days for 6 months during the study | SBP at 6 months=145.48 mmHg in control group and 130.10 mmHg in the intervention group.  DBP at 6 months=97.00 mmHg in control group and 88.83 mmHg in the intervention group |
| Mathews et al; [60] 2022 India | Outpatient clinic | RCT, 12 months | 210 (106/104) | Patients between 18 and 70 years of age, who were known hypertensive and on treatment for more than 2 years; 56 y* | Multifaceted face to face interview about their disease, medication, regimen, possible side effects, need for adherence, diet control, and exercise | Patient education | Every alternative month, over 12 months | Usual care | SBP at the end of study period=134.78 mmHg in control group and 125.81 mmHg in the intervention group.  DBP at the end of study period=85.273 mmHg in control group and 82.043 mmHg in the intervention group |
| Okoro et al; [61] 2022 Nigeria | Outpatient clinic | RCT, 12 months | 147 (73/74) | Hypertensive patients aged 18 to 85 years who were diagnosed with CKD at stages one to four; 52 y | BP home monitor, logbook, patient education, medication review, medication adherence and BP measurement reminder, telephone messages and calls | Patient education  Patient reminder | Visit at baseline, 6, 12 months, biweekly reminders | Participants in the usual care arm received the usual/conventional care offered by the hospitals which included hospital visits on appointment or a sick day, consultations with physicians, prescriptions for drugs and routine laboratory tests, review of diagnosis and medications, refilling of prescriptions by patients, and referrals | ITT SBP values at 12 months=135.9 mmHg in the intervention group and 140.0 mmHg in usual care (p=0.251).  ITT DBP values at 12 months=84.4 mmHg in the intervention group and 84.8 mmHg in usual care (p=0.840) |
| Paudel et al; [62] 2022 Nepal | Outpatient clinic | RCT, 4 months | 56 (28/28) | Participants aged 18 years or above, diagnosed with HT, taking at least one long-term antihypertensive Med for at least 6 months and having regular visits to the hospital for follow-up and/or Med refill; 56 y * | 1. A session of face-to-face counselling (approximately 15min) on hypertension and its risk factors;  2. Patient education on antihypertensive medication use and potential adverse effects; and  3. Lifestyle modification measures such as physical activity, salt intake, fruit and vegetable consumption, food consumption, smoking, body weight and stress | Patient education  Patient reminder | 15 min session; weekly telephone reminder; 2 face-to-face follow-up sessions in 2 months | Those in the control group received the usual care but not hospital pharmacist delivered individualised pharmaceutical service. As part of the usual care, patients had access to the pharmacist and usual counselling | SBP at second follow-up=125 mmHg in the intervention group and 130 mmHg in control group (p=0.008).  DBP at second follow-up=80 mmHg in the intervention group and 90 mmHg in control group (p=0.012) |
| Rohla et al; [63] 2022 Austria | Community pharmacy | Cluster RCT, 5 months | 352 (151/201) | Individuals aged 18 years or older with pharmacologically pre-treated HT and an AOBP above the threshold of 135/85 mmHg; 67 y | BP booklet (education, documentation); screening for uncontrolled HTN and referral to physician | Patient education  Healthcare provider feedback | Follow-up at 5, 10, 20 weeks | In the control group, patients received a BP record card which did not include background information about HTN. Follow-up visits after 5, 10 and 20 weeks were scheduled in accordance with the interventional group. Patients were encouraged to attend all four pharmacy visits before consulting their treating physician. Pharmacists in the control group were required to refer patients to their treating physician after 20 weeks of follow-up, in case 2 of 4 AOBP readings were above the threshold of 135/85 mmHg. Otherwise, referral was at the discretion of the pharmacists | SBP mean difference at the end of study period=5.8 mmHg (p=0.023).  DBP mean difference at the end of study period= 2.9 mmHg (p=0.049) |
| Torres-Robles et al; [64] 2022 Spain | Community pharmacy | Cluster RCT, 6 months | 502 (283/219) | Patients aged 18 years or older able to have a prescribed Med for HT; 64 y | Medication adherence management intervention:  1. Pharmacist interview to assess adherence to medications  2. Classification of patients as non-adherent  (non-intentional, intentional or combined) or adherent  3. Identification of barriers for medication adherence. Barriers could be practical, defined as gaps in knowledge or skills; or perceptual, namely those associated with the patient’s health beliefs and perceptions about the condition and their medications.  4. Intervention proposal using strategies tailored to the type of non-adherence and identified barriers  5. Application of the transtheoretical model of behavioral change by which the pharmacist elicited the patient’s readiness to change while discussing the proposed strategies.  6. Follow-up through monthly scheduled visits to review patient progress and provide feedback or new strategies to improve or maintain adherence.  7. Application of motivational interviewing principles and skills during the patient–pharmacist interaction | Patient education | Monthly scheduled visits over 6 months | Supply of medicines and medication-taking advice | Mean difference between intervention group and control group in SBP=-1.06 mmHg (p=0.48).  Mean difference between intervention group and control group in DBP=-1.70 mmHg (p=0.13) |
| Wang et al; [65] 2022 China | Outpatient clinic | RCT, 3 months | 80 (40/40) | Patients with DM2 and HT, aged between 18 and 65 years, admitted to the hospital for failing to control the blood glucose or BP, DM2and HT were well controlled according to the evaluation of physicians at discharge, received pharmaceutical care during hospitalization, and receiving antidiabetic and antihypertensive drugs after discharge; 42 y | 1. Consultations by clinical pharmacists after the visit with the physician.  2. Design of standardized science education materials.  3. Establishment of a “follow-up service,” WeChat exchange group and WeChat official account.  4. Continuous individualized follow-up. After discharge, the participants were followed by telephone every two weeks. | Patient education  Patient reminder | Over 3 months | Participants returned to the hospital every 2 weeks for regular clinical follow-up, including the prescription of hypoglycemic and antihypertensive drugs and the evaluation of blood sugar, BP, and other indexes. Nursing staff followed the participants by telephone once a month and recorded the participants’ blood sugar, BP, and medication compliance | BP control rate at 3 months (intervention group vs control group)=92.5% vs. 62.5% (p<0.001) |
| Gutierrez et al; [66] 2023 Philippines | Community health unit | RCT, 6 months | 417 (214/203) | Diagnosed hypertensive, receiving DOH  ComPack medicine, a PhilPEN program member for at least three  months, and prescribed Losartan, Amlodipine, and/or Metoprolol for  the next six months. Patient or primary caregiver have a  smartphone and internet access; 58 y | 1.) Personalized patient information material  2.) Medication reminder via smartphone  3.) Guide for pharmacists during patient counselling  4.) Formal communication channel for the patient and the pharmacist. | Patient education  Patient reminder | Daily medication reminder for 30 days, follow-up online counselling with pharmacist as needed | Monthly visit to the rural health unit for doctor’s consultation, one month supply of medication | SBP at 6 months 135.05 mmHg in intervention group, 132.20 mmHg in control group, p 0.1131  DBP at 6 months 86.79 mmHg in intervention group, 83.52 mmHg in control group, p 0.0011  BP controlled at 6 months 46.26% in intervention group, 63.55% in control group |
| Moral et al; [67] 2023 Spain | Community pharmacies | Cluster-RCT, 6 months | 388 (186/202) | Patients diagnosed with and pharmacologically treated for essential AHT,  who were literate, in full possession of their mental faculties and willing to be monitored  for 6 months, were included; 67 y | Health Education; Pharmacotherapy Follow-up; 24 h Ambulatory Blood Pressure Measurement for some patients | Patient education  Healthcare provider education | Two visits to the pharmacy  (beginning and end of the study) and received the  usual assistance in the community pharmacy for a pharmacologically treated hypertensive  patient | At least monthly over 6 months | BP controlled after intervention in 66.3% in control group, 85.8% in intervention group, p <0.001 |
| Moreira et al; [68] 2023 Brazil | Community pharmacy | RCT, 14 months | 322 (161/161) | Individuals aged ≥60 years, diagnosed with hypertension, used antihypertensive  drugs, and filled their rescriptions at a public community pharmacy in  Brazil; 67 y | Pharmaceutical anamnesis to identify prior health problems, listing medications in use and clinical manifestations of hypotension or uncontrolled hypertension, measuring the BP in the pharmacist’s office, and providing HBPM equipment.  The pharmacist’s clinical evaluation included a review of the drug treatment to identify specific requirements and problems regarding the  indication, effectiveness, and safety of the prescribed antihypertensive  therapy.  They forwarded a report with  the measurements to the GP. In this report, when necessary, the pharmacist made suggestions for improving the drug treatment. This involved deprescribing medications or adjusting the antihypertensive therapy, such as increasing the dose or adding a drug class. | Healthcare provider feedback  Patient education | Usual care including care provided by pharmacists (anamnesis to identify prior health problems, BP measurements in the pharmacist’s office, and were provided with an automatic BP monitor for  HBPM) | Follow-up after 45 days | Home SBP mean difference between control and intervention group -2.11 mmHg, p 0.29  Home DBP mean difference between control and intervention group -2.23 mmHg, p 0.07 |
| Mozu et al; [69] 2023 Ghana | Outpatient clinic | RCT, 7 months | 116 (57/59) | At least 18 years of age, medical diagnosis of hypertension, able to receive telephone calls; 63 y | Telepharmacy services: Monthly pharmacist-initiated telephone calls were scheduled. The patient follow-up mainly included the provision of pharmaceutical  care, answers to specific patient needs and encouragement of their own coping mechanisms in terms of lifestyle adjustments and risk factor reduction. The services offered included adherence counselling, patient  education, identification of care issues, blood pressure monitoring, initiating and promoting the practice of therapeutic lifestyles for the  control of hypertension. Individualized care with information based on  needs, readiness, and drive to learn were prioritized. A pharmaceutical care plan was drawn for each patient. Where physicians needed to make  changes to drug therapy, the recommended changes were communicated to the physician by the pharmacist. | Patient education  Healthcare provider feedback | Monthly over 6 months | Routine clinical care,  which included a single visit to the hypertension clinic either monthly or  bimonthly based on the level of blood pressure control or as determined on by the attending physician. Tele-pharmacy services were not offered. | SBP at 6 months 145.9 mmHg in control group, 134.8 mmHg in intervention group, p 0.001  DBP at 6 months 80.2 mmHg in control group, 79.5 mmHg in intervention group, p 0.198  BP controlled at 6 months 40.7% in control group, 66.1% in intervention group |
| ***Pharmacist collaborative care*** | | | | | | | | | |
| Earp et al; [70] 1982 United States | Outpatient clinic | RCT, 24 months | 218 (63/155) | Patients with a diagnosis of essential HT on a hypertensive Med which, in the previous two months, had been initiated, altered in some manner, or restarted after a drug-free hiatus; 48 y | Group 1 (N = 99) patients received both standard medical care and home visits, as well as had "significant others" whom they chose (usually family members) actively participate in both home visits and the BP monitoring process on a daily, or several times weekly, basis  Group 2 (N = 56) received standard medical care plus home visits over a period of 18 months from either public health nurses or specially trained pharmacists.  Team members: nurses | Patient education  Patient reminder | Over 18 months | Routine medical care | Supplementing routine care with periodic home visits produced well-controlled BP by an additional 21% of the patients, cutting in half the proportion who were not controlled by routine care alone |
| Bogden et al; [71] 1998 United States | Outpatient clinic | RCT, 6 months | 95 (49/46) | Uncontrolled hypertensive patients (BP≥150/95 mmHg; ≥140/90 if previous CVD or other CVD risk factors) taking antihypertensive Med or not; 55 y | Review of HT Med; Counseling of Med compliance; Review of laboratory data with physicians; Recommendation to physician regarding the least costly HT Med via patient chart.  Team members: physician | Patient education  Healthcare provider feedback | Over 6 months, 30 minutes per session | Same medical care except for the coordinated input from the pharmacist (standard medical practice at the Queen Emma Clinic). This included patient access to a pharmacy clerk to answer questions about medication. Unlike the intervention arm, access to the pharmacy clerk in the control arm needed to be initiated by the patient | SBP declined an average of 23 mmHg in the intervention arm and 11 mmHg in the control arm (p<0.01).  DBP declined an average of 14 mmHg in the intervention arm and 3 mm Hg in the control arm (p< 0.001) |
| Borenstein et al; [72] 2003 United States | Outpatient clinic | RCT, 12 months | 197 (98/99) | Uncontrolled hypertensive patients (BP≥140/90 mmHg; BP≥160/90 mmHg if age≥65 years); 62 y | Measurement of BP; Patient interview related to HT Med, compliance, and lifestyle; Education of dietary and lifestyle; Recommendation to physician regarding Med changes via telephone call.  Team members: physician and nurse | Patient education  Healthcare provider feedback | As often as deemed necessary by the clinical pharmacist until BP control | No intervention | At 12 months, reductions in SBP from baseline=22 mmHg for the intervention group (p<0.01) and 11 mmHg for the control group (p<0.01). The greater reduction of 10 mm Hg in systolic BP observed in intervention VS control patients was statistically significant (p<0.01).  This difference persisted after adjustment for baseline blood pressure.    At 12 months reductions in DBP from baseline= 7 mmHg for the intervention group (p<0.01) and 8 mmHg for the control group (p<0.01). The between-group difference in DBP was not statistically significant (p=0.53) |
| Carter et al; [73] 2008 United States | Outpatient clinic | cluster- RCT, 9 months | 179 (101/78) | Uncontrolled hypertensive patients (BP 145- 179/95-109 mmHg; BP 135-179/85-109 mmHg if DM) taking antihypertensive Med or not; 61 y | Patient interview related to Med; Verbally or written recommendation to physician regarding HT Med changes; Recommendation of Med compliance aids if necessary.  Team members: physician and nurse | Healthcare provider education | Pharmacists were encouraged to attend each clinic visit (2, 4, 6, and 8 months), and they were encouraged to initiate additional visits or telephone contact if BP remained uncontrolled | Patients in both groups were given written information on hypertension. The research nurses encouraged all patients to follow the lifestyle modifications. Patients were also made aware of their goal BP level | 24-hour BP effect with a mean difference (control  group minus the intervention group) of -8.8 mmHg (95%CI= -5.0 – -12.6) in SBP and -4.6 mm Hg (95%CI=-2.4 – -6.8) in DBP |
| Hunt et al; [74] 2008 United States | Outpatient clinic | RCT, 12 months | 463 (230/233) | Uncontrolled hypertensive patients (BP≥160/100 mmHg); 68 y | Review of Med and lifestyle habits; DRPs assessment; Written recommendation to physician regarding HT Med selection, dosage, and changes.  Team members: physician | Patient education  Healthcare provider feedback | Scheduled follow-up appointments as judged necessary | Subjects allocated to usual care were instructed to continue their normal schedule of medical care. The primary care physician was also provided a list of subjects allocated to usual care. Out of concern, subjects with an entry BP≥180/110 mmHg, based on last measurement in the electronic medical record were scheduled for an appointment with their primary care provider if a future appointment did not already exist | ITT (includes last value carried forward) mean (SD) SBP=142 mmHg for intervention and 148 mmHg for usual care (p=0.002).  ITT (includes last value carried forward) mean (SD) DBP=77 mmHg for intervention and 80 mmHg for usual care (p=0.003) |
| McLean et al; [75] 2008 Canada | Community pharmacy | RCT, 6 months | 227 (115/112) | Adult patients with DM and with BP>130/80 mmHg taking insulin or oral hypoglycemic Med for >6 months; 65 y | Pharmacist-nurse team including:  1. Patient education and counseling regarding cardiovascular risk reduction;  2. Distribution of HT education pamphlet and wallet card documenting recorded patient BP measures faxed to physicians;  3. Patient's risk factors, current Med and BP measures with any suggestions for further management based on guidelines faxed to physicians.  Team members: nurse | Patient education  Healthcare provider feedback | 6 weeks intervals, over 24 weeks | Patients randomized to usual care received the same BP wallet card with their BP measures documented, a pamphlet on diabetes, and general diabetes counseling from the nurse or pharmacist. Usual care patients received telephone follow-up at 12 weeks and no other follow-up until the in-person close-out visit at 24 weeks. Neither of these visits entailed any therapeutic advice to the usual care patients and were merely for the collection of end point data | The mean between-group difference in SBP was 5.6 (SE=2.1) mmHg (p=0.008) |
| Santschi et al; [76] 2008 Switzerland | Outpatient clinic and Community pharmacy | Cluster- RCT, 12 months | 68 (34/34) | Uncontrolled hypertensive patients (BP≥140/90 mmHg) taking antihypertensive Med; 66 y | Monitoring and support Med adherence with electronic device (adherence report), Discussion with GP and physician if necessary.  Team members: GP and physician | Patient reminder  Patient education | After enrolment (baseline visit), patients in both groups were seen at four scheduled visits: 2, 4, 6 and 12 months in the  physician's office | Patients in the usual care group received their antihypertensive treatment as usual from their community pharmacist without any special effort being made to improve patient's adherence | SBP at 12-month visit=148.6 mmHg in the intervention group and 154.1 mmHg in usual care (p=0.21).  DBP at 12-month visit=87.2 mmHg in intervention group and 84.5 mmHg in usual care (p=0.40) |
| Carter et al; [77] 2009 United States | Outpatient clinic | Cluster- RCT, 6 months | 402 (192/210) | Uncontrolled hypertensive patients (BP 140-179/90-109 mmHg; BP 130-179/80-109 mmHg if DM) taking 0 to 3 antihypertensive Med; 58 y | Assessment and adjustment of HT med approved by physicians; assessment of BP; verbally drug recommendations to physicians; education to physician if necessary.  Team members: physician | Healthcare provider education  Healthcare provider feedback | Pharmacists were encouraged to assess medications and BP at baseline and at 1 month and by telephone at 3 months and more frequently if necessary | Clinical pharmacists at control sites abstained from providing care for study patients but continued to answer general treatment questions from physicians | Mean difference in 24-hour BP= −10.3 (−23.7 to 3.1) mmHg for SBP and −3.1 (−9.0 to 2.8) mmHg for DBP |
| Rinfret et al; [78] 2009 Canada | Outpatient Clinic | RCT, 5 months | 223 (111/112) | Patients aged ≥ 18 years with an office diagnosis of HT who filled their prescriptions at one of the participating pharmacies; 56 y | Educational booklet, a digital home BP monitor, a logbook and access to a telephone-linked IT-supported management program  Team members: physicians, nurses | Patient reminder | Monthly reports | Control subjects  received usual care and educational materials | Change in average 24h SBP=-4.8 mmHg (p=0.001).  Change in average 24h DBP=-2.1 mmHg (p=0.007) |
| Edelman et al; [79] 2010 United States | Outpatient clinic | RCT, 12  months | 239 (133/106) | Patients with uncontrolled DM (HbA1c>7.5%) and HT (BP>140/90 mmHg) taking Med for DM; 62 y | At each group medical clinics session (comprising 7 to 8 patients with the care team):  1. Med review from medical records by pharmacist and physician;  2. Reviewing of BP and home blood glucose readings by pharmacist and physician;  3. Development of individualized plan for Med or lifestyle management with pharmacist and physician;  4. Adjustment of Med by pharmacist and physician and report to primary care providers; 5. Patient education and counseling related to Med and lifestyle.  Team members: physician and nurse | Patient education  Healthcare provider feedback | The groups met every 2 months (7 visits over 12 months). Sessions lasted 90-120 minutes; | Patients in the usual care group received no active intervention | Mean difference between groups in SBP= -7.3 mmHg (-12.8 to -1.7).  Mean difference between groups in DBP=-3.8 mmHg (-6.9 to -0.8) |
| Tobari et al; [80] 2010 Japan | Outpatient Clinic | RCT, 6 months | 132 (64/64) | Men and women 40–79 years of age, either taking anti- hypertensive Med under a stable regimen or treatment-naïve with SBP ranging from 140–179 mmHg and/or DBP ranging from 90-99 mmHg; 62 y | Patient education (bold messages which translated into individual goals, e.g., restriction of miso soup to one bowl or less per day and reducing the size of the evening meal). Patients with body mass index (BMI) ≥25 kg/m2 and those who walked <30min/day, were advised to record their body weights and total duration of physical activity daily. Recommendations to physician (medication changes)  Team members: physicians | Patient education  Healthcare provider feedback | 15-min sessions of monthly individual counseling for  6 months | Pharmacist counseling at baseline (20 min, orientation about the program, education about hypertension, practice in use of home BP device). Similar follow-up protocol, but the pharmacist’s monthly sessions and reports to the physician were omitted | Estimated difference between groups for SBP change at 6 months=-1.9 mmHg (-6.1 to +2.3)  Estimated difference between groups for DBP change at 6 months=-0.7 mmHg (-3.4 to +1.9) |
| Weber et al; [81] 2010 United States | Outpatient Clinic | Cluster RCT, 6 months | 175 (100/75) | Men or women aged 21 to 85 years with uncontrolled HT (clinic SBP average of the last 2 readings between 145- and 179-mm Hg or a DBP of 95 to 109 mm Hg; SBP between 135 to 179 mm Hg or DBP of 85 to 109 mm Hg if patients had DM), receiving treatment with 0 to 3 antihypertensive agents with no changes to their regimen within the past 4 weeks; 60 y | Reviewed patient data obtained by the research nurse and then interviewed the patient. Patient factors that might impede achieving the goal BP; the patients’ current treatment strategies compared with clinical guidelines; discussed treatment recommendations with the patients’ physicians  Team members: physicians | Healthcare provider feedback | 6 visits over 9 months (at baseline, 2, 4, 6, 8, and 9 months) | Patients in both groups had, structured study visits with a research nurse at baseline and at 2, 4, 6, 8, and 9 months. The clinic BP values were provided to the primary care provider for patients in the usual care group, and follow-up interventions were left to the discretion of the primary care providers | 24-hour SBP at end of study= 121.4 mmHg in the intervention group and 130.5 mmHg in control group (p=0.001)  24-hour DBP at end of study=69.2 mmHg in intervention group and 73.7 mmHg in control group (p=0.001) |
| Albsoul-Younes et al; [82] 2011 Jordan | Outpatient Clinic | RCT, 6 months | 266 (130/136) | Patients with uncontrolled HT, receiving 0-3 antihypertensive drugs with no change in the regimen or dose within the past 3 months; 57 y | Noted medication history, answered questions, encouraged compliance, instructions in self-monitoring of BP, advice on tobacco habits, healthy diet, educational materials, explained BP goals, recommendation of least costly and most effective antihypertensive drug choice to physician  Team members: physicians | Patient education  Healthcare provider feedback | Monthly, over 8 weeks, for 20-30 minutes | Monthly visits with pharmacist for data collection, no recommendations. Medical care by same physician team as intervention group | The mean reduction in SBP and DBP was significantly higher in the intervention group.  SBP mean reduction =10.6 (SD=13.5) in the control group and 16.1 (SD=14.6) in the intervention group (p =0.002).  DBP mean reduction=7.17(SD=13.11) in the control group and 10.5 (SD=12.9) in the intervention group (p=0.04) |
| Cohen et al; [83] 2011 United States | Outpatient Clinic | RCT, 6 months | 99 (50/49) | Patients with a diagnosis of DM2; HbA1c > 7.0%, LDL > 100 mg/dL (2.59 mmol/L) or LDL > 70 mg/dL (1.81 mmol/L) for those with coronary artery disease, and BP > 130/80 mm Hg, each documented at least once in the medical records in the 6 months before enrollment; 69 y | Education; behavioral and pharmacologic interventions for hypertension, hyperlipidemia, and hyperglycemia and tobacco use  Team members: nurses, dietitians, physical therapists | Patient education | 4 once-weekly 2-hour sessions, followed by 5 monthly booster session. The booster sessions lasted 90 minutes. | Standard of care. visits once every 4 months on average. The primary care providers have access to the same electronic medical record, which contains clinical reminders, computer-based references, drug formulary information, and referral services to diabetes self-management education, nutrition, physical therapy, and the weight loss program MOVE! | SBP at the end of study period=−9.19 (−14.95 to −3.43) in the intervention group and −0.80 (−5.61 to 4.02) in the control group |
| Santschi et al; [84] 2011 Canada | Community  pharmacy | Cluster RCT, 6 months | 89 (48/41) | Outpatients with HT (BP ≥130/80 mmHg) and CKD; 72 y | Pharmacists attended a communication-network program called ProFiL.  1. 3-h training workshop  for community pharmacists including lecture presentations on clinical presentations of CKD, management of DRPs among CKD outpatients, presentations of the program and clinical tools, and discussion of two real clinical cases;  2. Communication network to facilitate the transfer of clinical information (laboratory test results and medications documented by the nephrologist) between the pre-dialysis clinic and community pharmacists;  3. Pharmaceutical consultation service by hospital pharmacists with expertise in nephrology  Team members: physicians, nurses | Healthcare provider education  Healthcare provider feedback | 3-h training workshop for community pharmacists; 6 months follow-up | Usual care pharmacists did not have access to the ProFiL program and were asked to provide usual care | Adjusted change from baseline to 6 months in SBP=-11.6 mmHg (p=0.021).  Adjusted change from baseline to 6 months in DBP=-2.6 mmHg (p=0.348) |
| Svarstad et al; [85] 2013 United States | Community  pharmacy | Cluster RCT, 12 months | 576 (276/300) | Black patients aged 18 years or older, having one or more BP prescriptions with a mean BP of 140/90 mm Hg or more; 53 y | 1. Clinical toolkits for pharmacists and technicians: modified Brief Medication Questionnaires BMQs and other patient self-report tools, simple algorithms for addressing identified barriers, structured tools for faxing feedback to physicians, validated BP monitors and portable furniture for a semiprivate BP station.  2. Take-home toolkit for each patient (wallet card BP log,  medication box, leaflet, pedometer)  3.7-hour joint training session for intervention teams using multiple methods.  4. Participating corporations were contracted by the study director to implement the 6-month  intervention  Team members: pharmacy technicians | Patient education  Healthcare provider feedback | 6 monthly visits over 6 months | Control participants received patient information only, including a 14-page guide for lowering blood pressure, pamphlet about hypertension in black patients, cards showing their blood pressure at baseline and follow-up interviews, and instructions to seek immediate medical care for a blood pressure value greater than 210/115 mm Hg at 6- or 12-month follow-up. training | Compared with control participants, intervention participants achieved a net reduction of 7.31 mmHg in SBP (p< 0.001) and 2.95 mmHg in DBP (p=0.01) |
| Hirsch et al; [86] 2014 United States | Outpatient Clinic | RCT, 9 months | 667 (328/339) | Patients aged 18 ≥ years, diagnosed with HT, with the most recent BP measurement of ≥140/≥90 mm Hg (≥130/≥80 mm Hg if diagnosed with DM), on at least 1 antihypertensive Med; 68 y | Assessed the patient’s knowledge of hypertension and current treatment and reviewed current treatment goals, self-monitoring behavior, medical and medication history, and current medications. Helped the patient to set individual BP goals, reviewed and/or ordered laboratory tests, adjusted the antihypertensive-medication regimen. Each visit was documented in the electronic medical records system and routed to the patient’s primary care providers  Team members: physicians | Healthcare provider feedback | Patients were scheduled for four 30-minute pharmacist visits (baseline, 3, 6, and 9 months), independent of primary care providers visits, and as needed for follow-up with the pharmacist (additional clinic visit or via phone). The intervention was to be a limited time (9 months) of intensive medication therapy management, after which a patient would return to the primary care provider for the treatment of hypertension | Usual-care patients were not contacted but continued to see their primary care providers. In the intervention group, the pharmacist measured the BP at the beginning of each study visit, as was standard practice for all internal medicine clinic patients, whereas the nursing staff measured BP in the usual-care patients | Mean changes in ITT population in SBP= -5.2 mmHg in intervention group and -1.7 mmHg in usual care (p=0.22).  Mean changes in ITT population in DBP= -2.5 mmHg in the intervention group and -0.3 mmHg in usual care, (p=0.27) |
| Leiva et al; [87] 2014 Spain | Outpatient Clinic | RCT, 12 months | 223 (114/109) | Patients aged 18–80 years with HT (SBP ≥140 mmHg and/or average DBP ≥90 mmHg on two visits 2 weeks apart; in patients with DM2 or renal failure BP ≥130/80 mmHg), taking antihypertensive Med drugs with uncontrolled BP; 66 y | 1. Motivational interview;  2. Pillbox reminder: a 7-day pillbox organizer was provided to each patient and its use explained;  3. Family support;  4. BP measurements and AHT reminder forms;  5. Simplification of dosing regimen  Team members: nurses | Patient reminder  Healthcare provider feedback | 3 interventional visits. The first follow-up visit after 1 month consisted of a motivational interview (25 minutes), simplification of dosing regimen, and instructions on a pill reminder (5 minutes), self-recording of BP (5 minutes), and family support (5 minutes). The second and third visits after 3 and 9 months, respectively, consisted of a motivational interview (25 minutes), check of self-recorded BP (5 minutes), and family support (5 minutes) | Patients randomized to the control group did not receive any change in their care. However, they were contacted at baseline and at 12 months and asked to complete the same measurements and interviews as the intervention group | Adjusted analysis for change between baseline value and the 12 months value for SBP= -5.1 mmHg in the intervention group and -2.1 mmHg in control group (p=0.219).  Adjusted analysis for change between baseline value and the 12 months value for DBP= -1.19 mmHg in the intervention group and -0.16 mmHg in control group (p=0.515) |
| Carter et al; [88] 2015 United States | Outpatient Clinic | Cluster RCT, 24 months | 418 (224/194) | Patients with uncontrolled HT; 50% with DM or CKD.; 60 y | Medical record review by the pharmacist and a structured interview with the subject, including:  1. A medication history;  2. An assessment of knowledge of BP medications, dosages and timing, and potential side effects; and  3. Other barriers to BP control (e.g., side effects and nonadherence)  Team members: physicians | Patient education  Healthcare provider feedback | Telephone call at 2 weeks, structured face-to-face visits at baseline, 1, 2, 4, 6, and 8 months and additional visits if BP remained uncontrolled | Usual care | Adjusted mean difference SBP=-6.1 mmHg (p=0.002).  Adjusted mean difference DBP=-2.9 mmHg (p=0.005) |
| Qudah et al; [89] 2016 Jordan | Outpatient Clinic | RCT, 6 months | 52 (27/25) | Patients 18 years or older, receiving hemodialysis, with untreated HT (weekly average home BP above 135/85 mmHg and not receiving any antihypertensive Med) or taking antihypertensive Med; 39 y | Physician-pharmacist collaborative care; Recommendations to physician; Educational material to patient; Patient counselling including education, BP goals, adverse drug reaction; Home BP readings.  Team members: physicians | Patient education  Healthcare provider feedback | Usually met 2-3 times during follow-up. | Patients in the control arm received the same medical care as those in the intervention arm except for the coordinated input from the pharmacist. Baseline weekly home BP readings were obtained from patients in the control arm on enrollment and at the end of the study without providing any recommendations or interventions | Mean difference between two study arms in average home SBP=-14.4 mmHg, (p=0.009)  Mean difference between two study arms in average home DBP=-3.9 mmHg (p=0.238) |
| Scala et al; [90] 2018 Italy | Outpatient Clinic | RCT, 12 months | 164 (84/80) | Adults aged 18 or over with an established medical diagnosis of arterial HT and with uncontrolled BP (SBP>140mmHg; DPB>90mmHg; SBP>130mmHg; DPB>80mmHg with DM or CKD) on established antihypertensive Med for at least 6 months; 58 y | Educational support: perceived risk of and knowledge about hypertension, adverse effects of drug therapy, memory, weight, exercise, diet, smoking, and alcohol use.  Team members: physicians | Patient education  Patient reminder | Telephone calls/interviews approximately every 2 months for 1 year | The usual care consisted of the routine care provided by multidisciplinary Health Care Team for treatment of hypertension, including face-to-face meetings with the pharmacist who discussed drug therapy as prescribed by the physician during the visit and lifestyle modifications with the patients | SBP at 12 months= 135.5 mmHg in the intervention group and 147.9 mmHg in control group (p=0.001).  DBP at 12 months= 86.0 mmHg in the intervention group and 90.6 mmHg in control group (p=0.001) |
| Peralta et al; [91] 2020 United States | Outpatient Clinic | Cluster RCT, 12 months | 1220 (616/604) | Patients aged 18–80 years, with documented HT in the past 5 years, and had a primary care visit with an eligible primary care provider in the past 18 months along with no diagnosis of CKD; 68 y | CKD screening, education, plus pharmacist co-management  Team members: physicians | Patient education  Healthcare provider feedback | 1 year follow-up | Patients who were randomized to usual care were not systematically screened for CKD | Change in SBP from baseline after ITT analyses= -1 mmHg in usual care and -2 mmHg in the intervention group (p=0.49).  Change in DBP from baseline after ITT analyses= -2 mmHg in usual care and -1 mmHg in the intervention group (p=0.72) |
| Santschi et al; [92] 2021 Switzerland | Outpatient Clinic and Community pharmacy | RCT, 12 months | 89 (43/46) | Patients with uncontrolled HT (daytime ambulatory BP≥135/85 mmHg or officeBP≥140/90 mmHg over at least two consecutive visits) aged 18 years or more, taking at least 1 antihypertensive Med; 61 y | Team-based care:  1. A structured individual intervention conducted by trained nurses and community pharmacists.  2. BP measurement, assessment, and counseling  about lifestyle and medication adherence and, health education concerning treatment and disease.  3. Following each 6-week visit, a summary report (BP measures,  medication adherence and lifestyle assessment) with  recommendations were prepared by the nurse and the pharmacist for the physician who adjusted antihypertensive therapy accordingly  Team members: nurses | Patient education  Healthcare provider feedback | Every 6 weeks (at baseline, 6-, 12-, 18-week) during the 6-month of follow-up | Patients allocated to usual care group received routine care by their habitual physician without any specific nurse or community pharmacist intervention. They attended schedule visits at baseline, 6 and 12 months of follow-up, where ABPM was taken. | Mean between group difference at 12 months in SBP= -7 mmHg (p=0.01).  Mean between group difference at 12 months in DBP=-2 mmHg (p=0.42) |
| Lau et al; [93] 2022 Canada | Home | RCT, 12 months | 92 (47/45) | Adults aged ≥65 years with a diagnosis of HT (self-reported or documented prescription of an anti-hypertensive Med); 80 y | Home BP telemonitoring; pharmacist-led case management (behavioral counselling, teach BP self-monitoring; encourage medication adherence; review BP; remind home BP monitoring; adjust medications)  Team members: physicians | Patient education  Patient reminder | 12 months follow-up; case manager contacts at baseline and every 3 months or more frequently at their own discretion | Enhanced usual care: Home BP monitoring equipment and training only | Adjusted difference in 24 h-ABPM in SBP= -1.6 mmHg (p =0.256).  Adjusted difference in 24 h-ABPM in DBP=-1.1 mmHg (p 0.101) |
| Naqvi et al; [94] 2022 United States | Outpatient Clinic | RCT, 3 months | 45 (23/22) | Patients with an acute ischemic or hemorrhagic stroke discharged home after hospitalization with a diagnosis of HT and ability to provide consent;64 y | Telehealth After Stroke Care: a tablet and monitor to wirelessly transmit BP data to the electronic health record, with telenursing support, tailored infographics to explain BP readings, and pharmacist visits.  Team members: primary care nurse practitioner (NP), physicians | Patient education  Patient reminder | Telehealth visits after discharge were scheduled with the primary care nurse practitioner at 1 to 2 weeks (±5 days), pharmacist at 4 and 8 weeks (±5 days), and physician at 6 and 12 weeks (±5 days) | Participants randomized to usual care received a transitional care management visit at 1 to 2 weeks post-discharge with primary care. In addition, they received stroke physician video visits at 6 and 12 weeks. Although not established standard of care, these are considered best practices | SBP was 18.4 mmHg lower from baseline in the intervention arm (−32.5 to −4.4 mmHg, p=0.01) |
| Nguyen-Huynh et al; [95] 2022 United States | Outpatient Clinic | Cluster RCT, 48 months | 1475 (346/1129) | Adults 18 years or older with BP of at least 140/90 mm Hg of self-reported Black or African American race; 60 y | Enhanced pharmacotherapy monitoring protocol: delivered by a research nurse coordinator and a pharmacist; medical assistant BP check visits, optimize thiazide diuretic dosing, increase prescribing of spironolactone for resistant hypertension (i.e., receiving 2 antihypertensives). The research nurse also raised awareness through education on hypertension and the importance of BP control to reduce the risk of cardiovascular disease and stroke, identified potential barriers to controlling BP  Team members: nurse | Patient education | Intervention over 12 months | Free subsequent BP check visits, during which a medical assistant measured and recorded the patient’s BP using an automated BP cuff and reviewed their current BP medications. Findings were reviewed by the primary care physician or a local pharmacist, and any necessary adjustments to the BP medication regimen were made. | Mean SBP 48 months post-enrollment in enhanced group=135.1 mmHg, in usual care 135.1 mmHg  Mean DBP 48 months post-enrollment in enhanced group 73.41 mmHg, in usual care 74.49 mmHg |

Abbreviations: RCT: randomized controlled trial; HT: hypertension; Med: medication; BP: blood pressure; SBP: systolic blood pressure; DBP: diastolic blood pressure; ITT: intention-to-treat analyses; SE: standard error; GP: general practitioner; DRP: drug related problems; HbA1c: glycated haemoglobin; DM: diabetes mellitus; DM2: diabetes mellitus type 2; AOBP: automated office blood pressure; ABPM: ambulatory blood pression monitoring; CKD: chronic kidney disease CVD: cardiovascular; COPD: chronic obstructive pulmonary disease; LDL: low-density lipoproteins

*: The mean age was not reported and was computed using the age categories

**: age was not reported

**Supplemental Table S4.** Grading of Recommendations Assessment, Development and Evaluation (GRADE) assessment of the evidence for the effect of pharmacist interventions on systolic blood pressure (BP).

|  | **Outcome** | **Mean difference (95% CI) with pharmacist interventions** | **N participants (N studies)** | **Certainty (GRADE assessment)** |
| --- | --- | --- | --- | --- |
| All studies | Systolic BP | 5.3 mmHg lower (between 4.4 and 6.3 mmHg) | 27 057 (76 RCTs) | Low^a^ |
| High quality studies | Systolic BP | 4.8 mmHg lower (between 3.7 and 5.9 mmHg) | 21 339 (39 RCTs) | Moderate^b^ |

CI= confidence interval

^a^ downgraded to “Low” due to several studies at high risk of bias and possible publication bias

^b^ downgraded to “Moderate” due to several studies at high risk of bias

**Supplemental Figure S1.** Results of the risk of bias assessment using the revised Cochrane risk of bias (RoB 2) tool and visualised with the robvis tool. [96, 97]


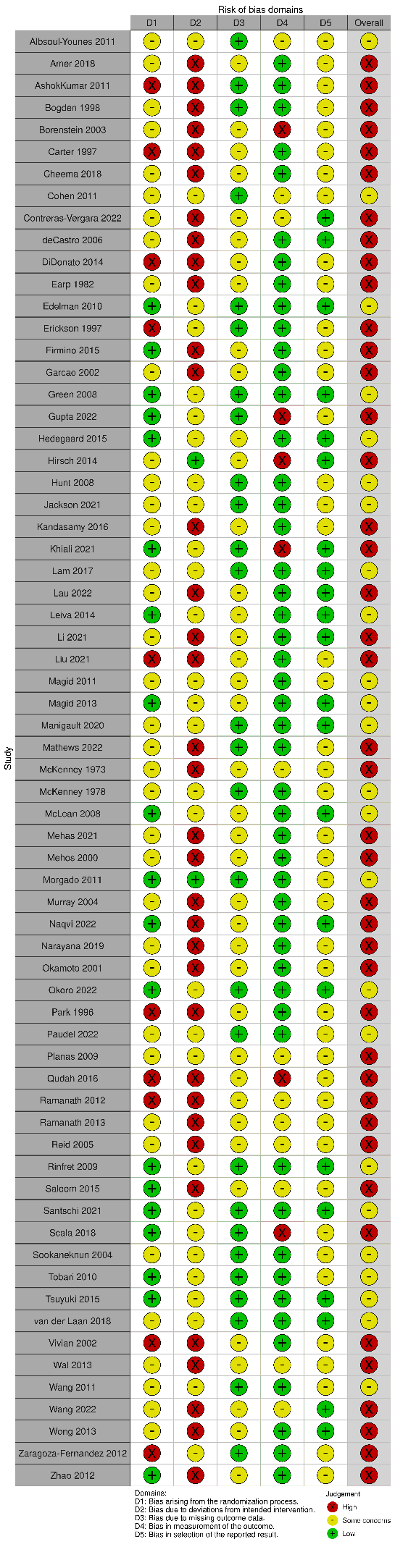


**Supplemental Figure S2.** Sensitivity analysis limited to relatively high-quality studies. Forest plot of the mean difference between pharmacist and usual care group in systolic blood pressure sorted by year of publication.


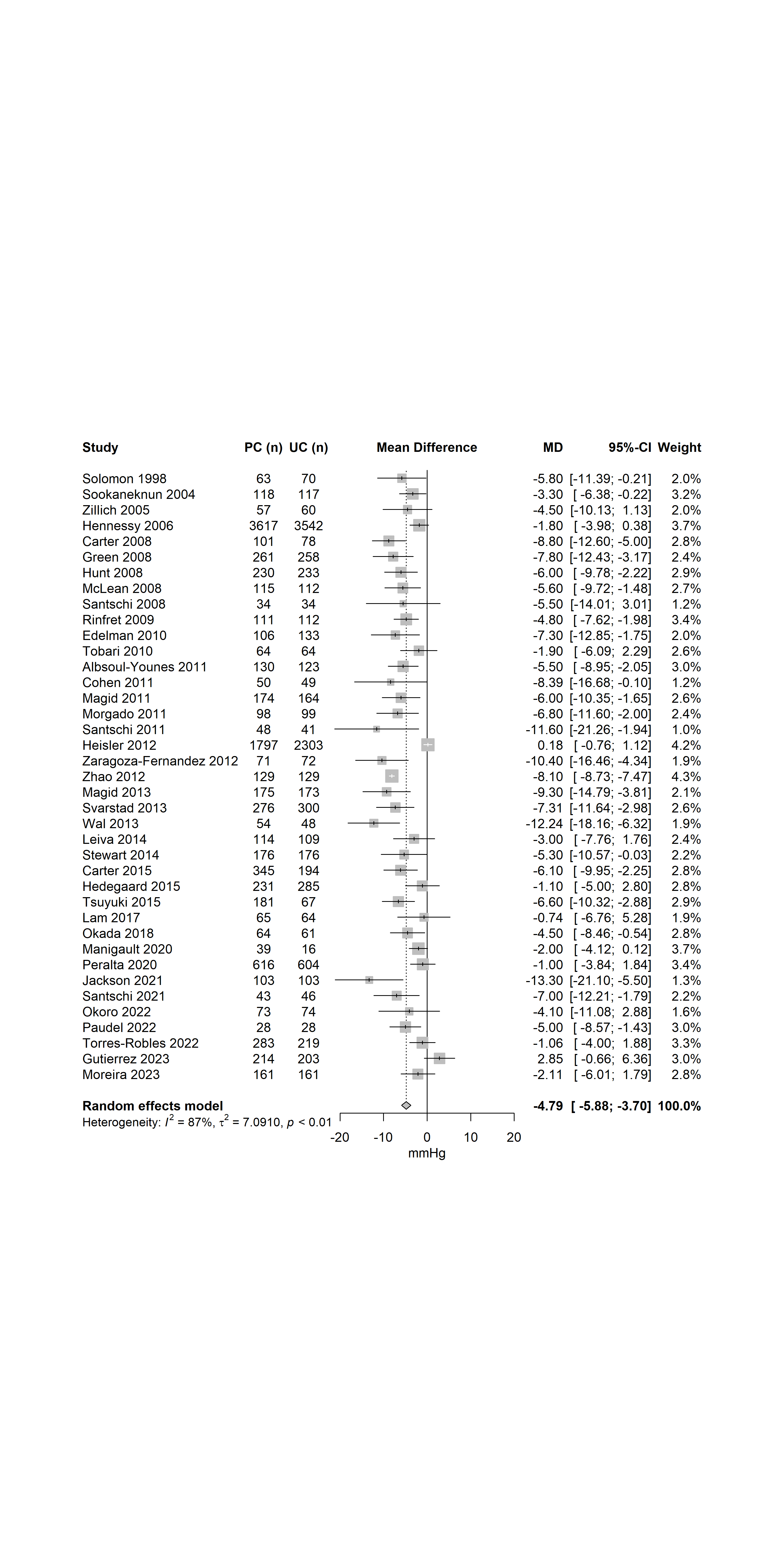


**Supplemental Figure S3.** Sensitivity analysis of relatively high-quality studies. Forest plot of the mean difference between pharmacist and usual care group in diastolic blood pressure sorted by year of publication.


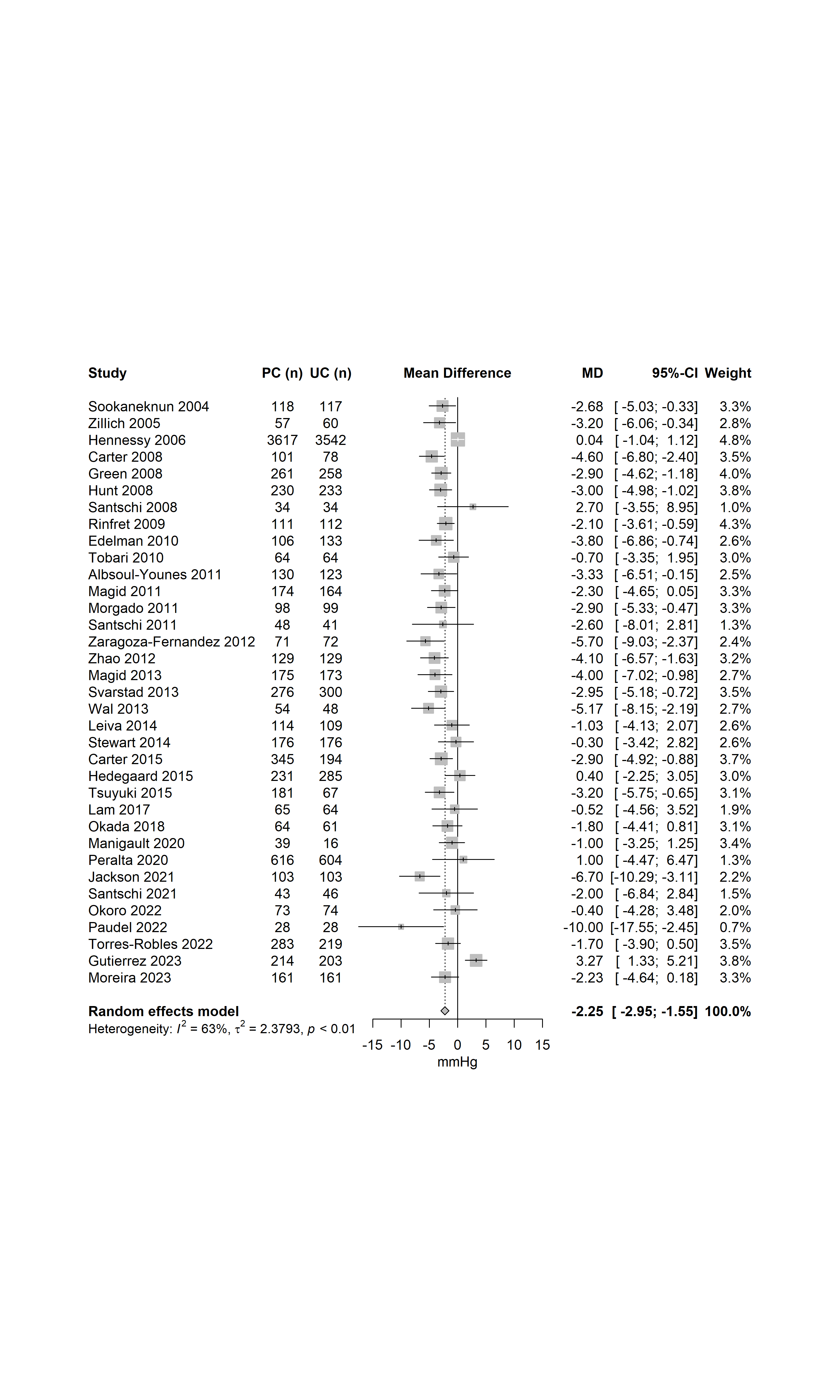


**Supplemental Figure S4.** Forest plot of the relative risk between pharmacist and usual care group in blood pressure control sorted by year of publication.


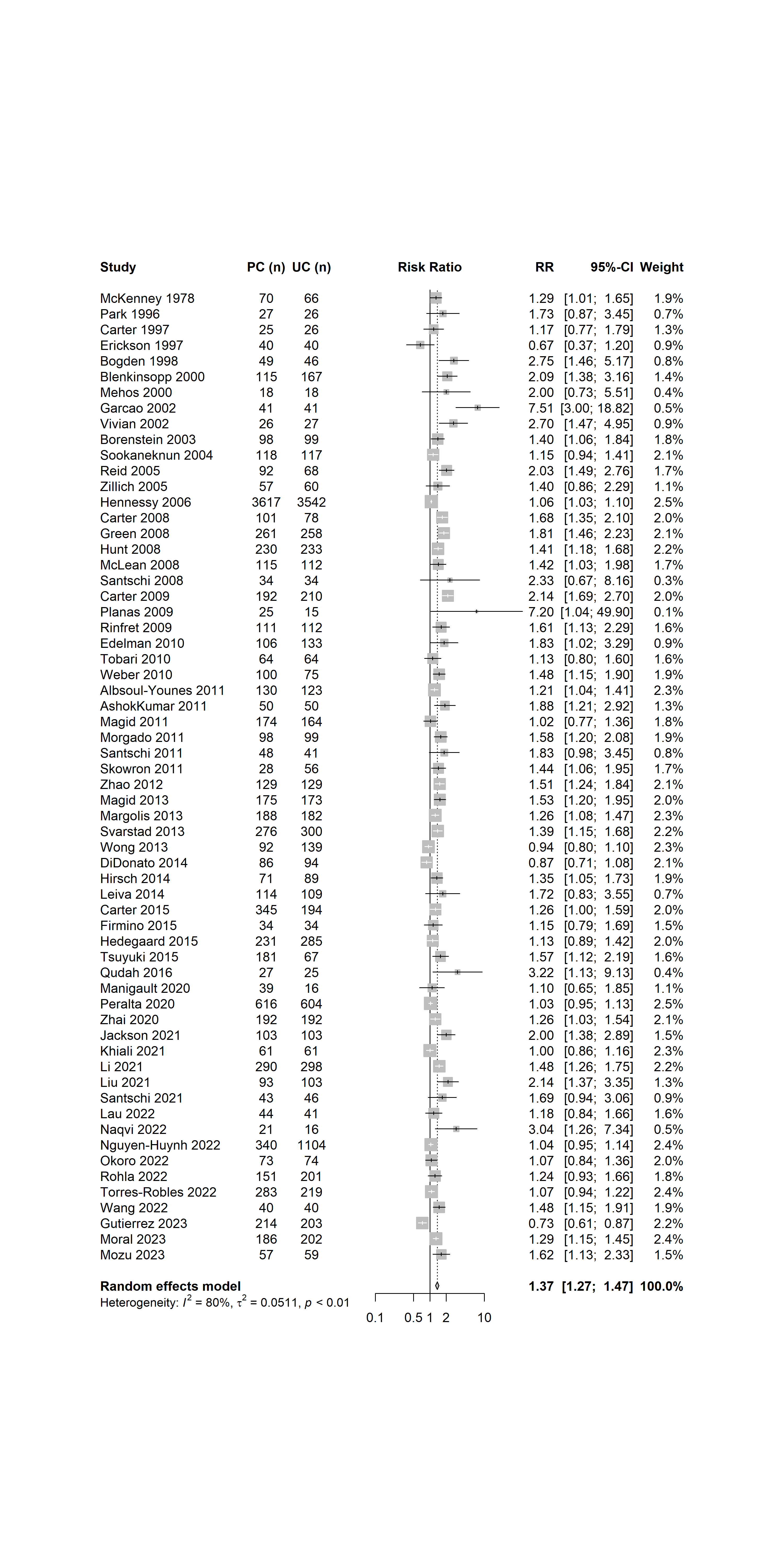


**Supplemental Figure S5.** Funnel plots to assess publication bias for systolic blood pressure (left panel) and diastolic blood pressure (right panel).


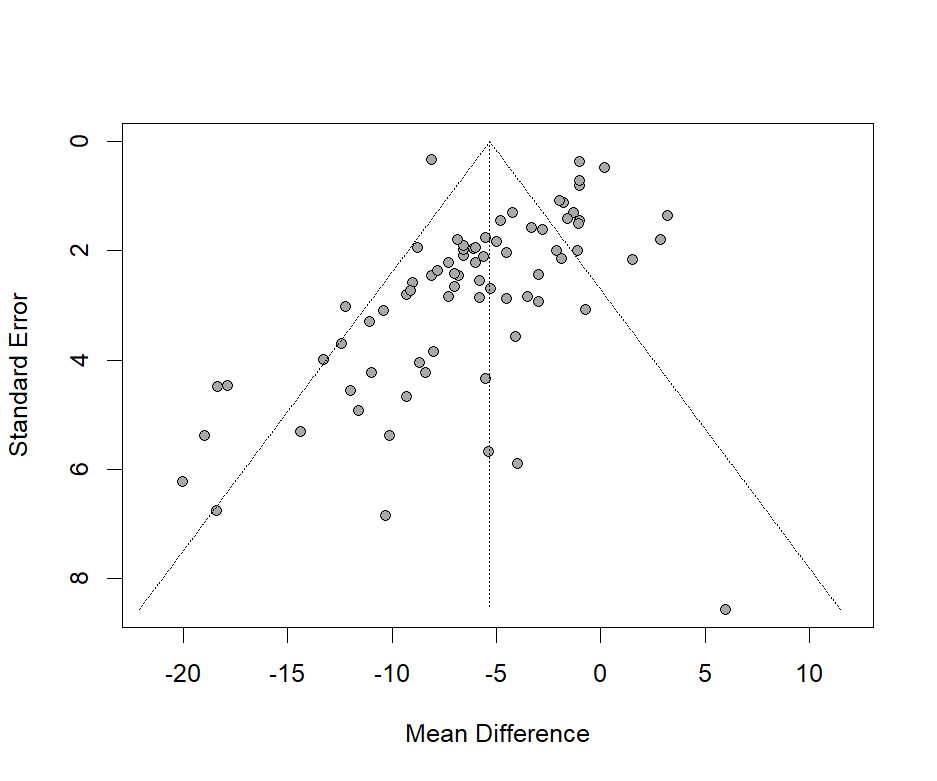

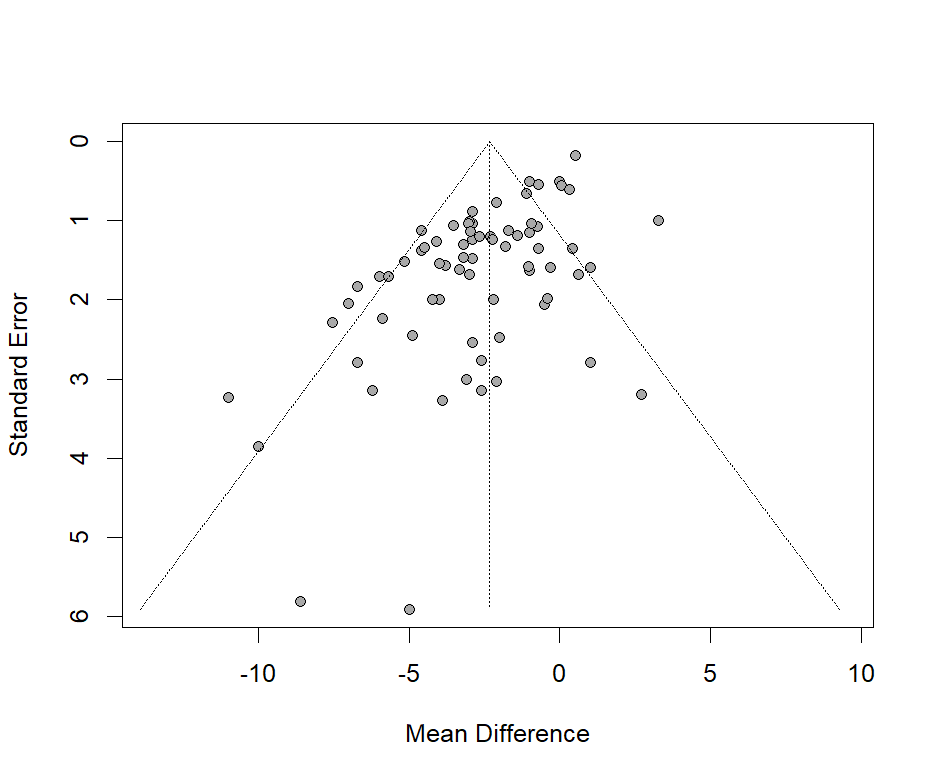


**Supplemental Figure S6.** Funnel plots to assess publication bias, excluding studies at high risk-of-bias for systolic blood pressure (left panel) and diastolic blood pressure (right panel).


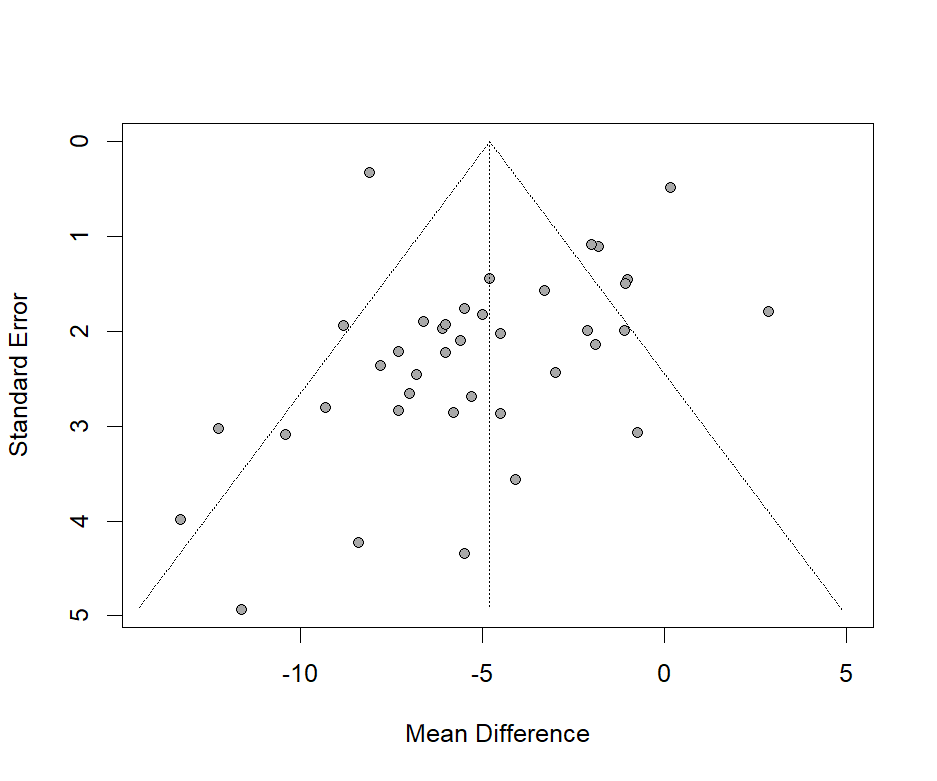

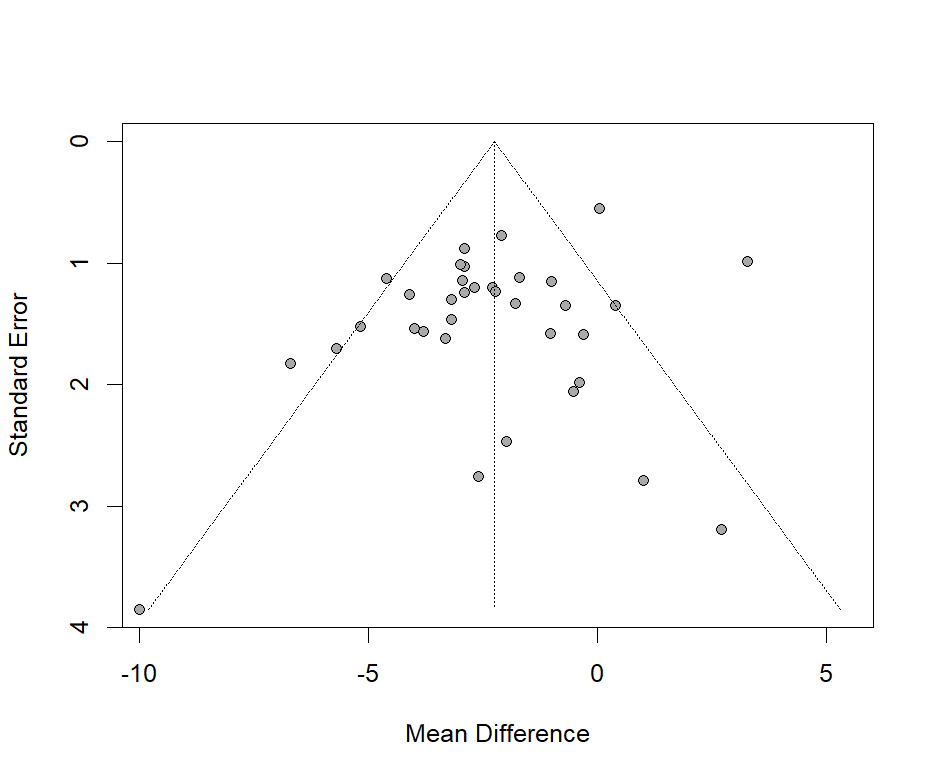


**REFERENCES**

| [1] | J. M. McKenney, J. M. Slining, H. R. Henderson, D. Devins and M. Barr, "The effect of clinical pharmacy services on patients with essential hypertension," *Circulation,* vol. 48, p. 1104–1111, 1973. |
| --- | --- |
| [2] | J. M. McKenney, E. D. Brown, R. Necsary and H. L. Reavis, "Effect of pharmacist drug monitoring and patient education on hypertensive patients," *Contemporary pharmacy practice,* vol. 1, p. 50–56, 1978. |
| [3] | J. J. Park, P. Kelly, B. L. Carter and P. P. Burgess, "Comprehensive Pharmaceutical Care in the Chain Setting: Drug therapy monitoring and counseling by pharmacists contributed to improved blood pressure control in study patients.," *Journal of the American Pharmaceutical Association (1996),* vol. 36, p. 443–451, 1996. |
| [4] | B. L. Carter, D. J. Barnette, E. Chrischilles, G. J. Mazzotti and Z. J. Asali, "Evaluation of hypertensive patients after care provided by community pharmacists in a rural setting," *Pharmacotherapy: The Journal of Human Pharmacology and Drug Therapy,* vol. 17, p. 1274–1285, 1997. |
| [5] | S. R. Erickson, R. Slaughter and H. Halapy, "Pharmacists' ability to influence outcomes of hypertension therapy," *Pharmacotherapy: The Journal of Human Pharmacology and Drug Therapy,* vol. 17, p. 140–147, 1997. |
| [6] | D. K. Solomon, T. S. Portner, G. E. Bass, D. R. Gourley, G. A. Gourley, J. M. Holt, W. R. Wicke, R. L. Braden, T. N. Eberle, T. H. Self and others, "Part 2. Clinical and economic outcomes in the hypertension and COPD arms of a multicenter outcomes study," *Journal of the American Pharmaceutical Association (1996),* vol. 38, p. 574–585, 1998. |
| [7] | A. Blenkinsopp, M. Phelan, J. Bourne and N. Dakhil, "Extended adherence support by community pharmacists for patients with hypertension: a randomised controlled trial," *International Journal of Pharmacy Practice,* vol. 8, p. 165–175, 2000. |
| [8] | B. M. Mehos, J. J. Saseen and E. J. MacLaughlin, "Effect of pharmacist intervention and initiation of home blood pressure monitoring in patients with uncontrolled hypertension," *Pharmacotherapy: The Journal of Human Pharmacology and Drug Therapy,* vol. 20, p. 1384–1389, 2000. |
| [9] | M. P. Okamoto and R. K. Nakahiro, "Pharmacoeconomic evaluation of a pharmacist-managed hypertension clinic," *Pharmacotherapy: The Journal of Human Pharmacology and Drug Therapy,* vol. 21, p. 1337–1344, 2001. |
| [10] | J. A. Garçao and J. Cabrita, "Evaluation of a pharmaceutical care program for hypertensive patients in rural Portugal," *Journal of the American Pharmaceutical Association (1996),* vol. 42, p. 858–864, 2002. |
| [11] | E. M. Vivian, "Improving blood pressure control in a pharmacist-managed hypertension clinic," *Pharmacotherapy: The Journal of Human Pharmacology and Drug Therapy,* vol. 22, p. 1533–1540, 2002. |
| [12] | M. D. Murray, L. E. Harris, J. M. Overhage, X.-H. Zhou, G. J. Eckert, F. E. Smith, N. N. Buchanan, F. D. Wolinsky, C. J. McDonald and W. M. Tierney, "Failure of computerized treatment suggestions to improve health outcomes of outpatients with uncomplicated hypertension: results of a randomized controlled trial," *Pharmacotherapy: The Journal of Human Pharmacology and Drug Therapy,* vol. 24, p. 324–337, 2004. |
| [13] | P. Sookaneknun, R. M. E. Richards, J. Sanguansermsri and C. Teerasut, "Pharmacist involvement in primary care improves hypertensive patient clinical outcomes," *Annals of Pharmacotherapy,* vol. 38, p. 2023–2028, 2004. |
| [14] | F. Reid, P. Murray and M. Storrie, "Implementation of a pharmacist-led clinic for hypertensive patients in primary care–a pilot study," *Pharmacy World and Science,* vol. 27, p. 202–207, 2005. |
| [15] | A. J. Zillich, J. M. Sutherland, P. A. Kumbera and B. L. Carter, "Hypertension outcomes through blood pressure monitoring and evaluation by pharmacists (HOME study)," *Journal of general internal medicine,* vol. 20, p. 1091–1096, 2005. |
| [16] | M. S. De Castro, F. D. Fuchs, M. Costa Santos, P. Maximiliano, M. Gus, L. Beltrami Moreira and M. B. Cardoso Ferreira, "Pharmaceutical care program for patients with uncontrolled hypertension: report of a double-blind clinical trial with ambulatory blood pressure monitoring," *American journal of hypertension,* vol. 19, p. 528–533, 2006. |
| [17] | S. Hennessy, C. E. Leonard, W. Yang, S. E. Kimmel, R. R. Townsend, A. G. Wasserstein, T. R. Ten Have and W. B. Bilker, "Effectiveness of a two-part educational intervention to improve hypertension control: a cluster-randomized trial," *Pharmacotherapy: The Journal of Human Pharmacology and Drug Therapy,* vol. 26, p. 1342–1347, 2006. |
| [18] | B. B. Green, A. J. Cook, J. D. Ralston, P. A. Fishman, S. L. Catz, J. Carlson, D. Carrell, L. Tyll, E. B. Larson and R. S. Thompson, "Effectiveness of home blood pressure monitoring, Web communication, and pharmacist care on hypertension control: a randomized controlled trial," *Jama,* vol. 299, p. 2857–2867, 2008. |
| [19] | L. G. Planas, K. M. Crosby, K. D. Mitchell and K. C. Farmer, "Evaluation of a hypertension medication therapy management program in patients with diabetes," *Journal of the American Pharmacists Association,* vol. 49, p. 164–170, 2009. |
| [20] | L. H. Jamieson, A. Scally and H. Chrystyn, "A randomised comparison of practice pharmacist-managed hypertension providing Level 3 Medication Review versus usual care in general practice.," *Journal of applied therapeutic research,* vol. 7, p. 77–86, 2010. |
| [21] | K. M. Ashok, J. Elayaraja, K. Shailaja and C. Ramasamy, "Improving medication adherence and clinical outcomes of hypertensive patients through patient counseling," *Res J Pharm Biol Chem Sci,* vol. 2, p. 231–241, 2011. |
| [22] | D. J. Magid, P. M. Ho, K. L. Olson, D. W. Brand, L. K. Welch, K. E. Snow, A. C. Lambert-Kerzner, M. E. Plomondon and E. P. Havranek, "A multimodal blood pressure control intervention in 3 healthcare systems.," *The American journal of managed care,* vol. 17, p. e96–103, 2011. |
| [23] | M. Morgado, S. Rolo and M. Castelo-Branco, "Pharmacist intervention program to enhance hypertension control: a randomised controlled trial," *International journal of clinical pharmacy,* vol. 33, p. 132–140, 2011. |
| [24] | A. Skowron, S. Polak and J. Brandys, "The impact of pharmaceutical care on patients with hypertension and their pharmacists," *Pharmacy practice,* vol. 9, p. 110, 2011. |
| [25] | J. Wang, J. Wu, J. Yang, Y. Zhuang, J. Chen, W. Qian, J. Tian, X. Chen, D. She and F. Peng, "Effects of pharmaceutical care interventions on blood pressure and medication adherence of patients with primary hypertension in China," *Clinical Research and Regulatory Affairs,* vol. 28, p. 1–6, 2011. |
| [26] | M. Heisler, T. P. Hofer, J. A. Schmittdiel, J. V. Selby, M. L. Klamerus, H. B. Bosworth, M. Bermann and E. A. Kerr, "Improving blood pressure control through a clinical pharmacist outreach program in patients with diabetes mellitus in 2 high-performing health systems: the adherence and intensification of medications cluster randomized, controlled pragmatic trial," *Circulation,* vol. 125, p. 2863–2872, 2012. |
| [27] | K. V. Ramanath, D. B. S. S. Balaji, C. H. Nagakishore, S. M. Kumar and M. Bhanuprakash, "A study on impact of clinical pharmacist interventions on medication adherence and quality of life in rural hypertensive patients," *Journal of Young Pharmacists,* vol. 4, p. 95–100, 2012. |
| [28] | M. P. Zaragoza-Fernandez, M. A. Gastelurrutia, M. Cardero and F. Martinez-Martinez, "Intensive two-month intervention on diet and lifestyle in uncontrolled hypertensive patients in a community pharmacy," *Latin Am J Pharm,* vol. 31, p. 727–733, 2012. |
| [29] | P.-X. Zhao, C. Wang, L. Qin, M. Yuan, Q. Xiao, Y.-H. Guo and A.-D. Wen, "Effect of clinical pharmacist’s pharmaceutical care intervention to control hypertensive outpatients in China," *Afr J Pharm Pharmacol,* vol. 6, p. 48–56, 2012. |
| [30] | D. J. Magid, K. L. Olson, S. J. Billups, N. M. Wagner, E. E. Lyons and B. A. Kroner, "A pharmacist-led, American Heart Association Heart360 Web-enabled home blood pressure monitoring program," *Circulation: Cardiovascular Quality and Outcomes,* vol. 6, p. 157–163, 2013. |
| [31] | K. L. Margolis, S. E. Asche, A. R. Bergdall, S. P. Dehmer, S. E. Groen, H. M. Kadrmas, T. J. Kerby, K. J. Klotzle, M. V. Maciosek, R. D. Michels and others, "Effect of home blood pressure telemonitoring and pharmacist management on blood pressure control: a cluster randomized clinical trial," *Jama,* vol. 310, p. 46–56, 2013. |
| [32] | K. V. Ramanath, K. R. Venkappa and others, "Study the impact of clinical pharmacist provided patient counseling on hypertension management in rural Indian population," *Archives of Pharmacy Practice,* vol. 4, p. 28, 2013. |
| [33] | P. Wal, A. Wal, A. Bhandari, U. Pandey and A. K. Rai, "Pharmacist involvement in the patient care improves outcome in hypertension patients," *Journal of research in pharmacy practice,* vol. 2, p. 123, 2013. |
| [34] | M. C. S. Wong, K. Q. L. Liu, H. H. X. Wang, C. L. S. Lee, M. W. M. Kwan, K. W. S. Lee, Y. Cheung, G. K. Y. Lee, D. E. Morisky and S. M. Griffiths, "Effectiveness of a pharmacist-led drug counseling on enhancing antihypertensive adherence and blood pressure control: a randomized controlled trial," *The Journal of Clinical Pharmacology,* vol. 53, p. 753–761, 2013. |
| [35] | K. L. DiDonato, K. R. Vetter, Y. Liu, J. R. May and D. M. Hartwig, "Examining the effect of a medication synchronization or an education program on health outcomes of hypertensive patients in a community pharmacy setting," 2014. |
| [36] | K. Stewart, J. George, K. P. Mc Namara, S. L. Jackson, G. M. Peterson, L. R. Bereznicki, P. R. Gee, J. D. Hughes, M. J. Bailey, Y. A. Hsueh and others, "A multifaceted pharmacist intervention to improve antihypertensive adherence: a cluster-randomized, controlled trial (HAPPy trial)," *Journal of clinical pharmacy and therapeutics,* vol. 39, p. 527–534, 2014. |
| [37] | P. Y. M. Firmino, T. O. Vasconcelos, C. C. Ferreira, L. M. Moreira, N. R. Romero, L. A. Dias, M. G. R. d. Queiroz, M. V. d. O. Lopes and M. M. d. F. Fonteles, "Cardiovascular risk rate in hypertensive patients attended in primary health care units: the influence of pharmaceutical care," *Brazilian Journal of Pharmaceutical Sciences,* vol. 51, p. 617–627, 2015. |
| [38] | U. Hedegaard, L. J. Kjeldsen, A. Pottegård, J. E. Henriksen, J. Lambrechtsen, J. Hangaard and J. Hallas, "Improving medication adherence in patients with hypertension: a randomized trial," *The American journal of medicine,* vol. 128, p. 1351–1361, 2015. |
| [39] | F. Saleem, M. A. Hassali, A. A. Shafie, N. Ul Haq, M. Farooqui, H. Aljadhay and F. U. D. Ahmad, "Pharmacist intervention in improving hypertension-related knowledge, treatment medication adherence and health-related quality of life: a non-clinical randomized controlled trial," *Health Expectations,* vol. 18, p. 1270–1281, 2015. |
| [40] | R. T. Tsuyuki, S. K. D. Houle, T. L. Charrois, M. R. Kolber, M. M. Rosenthal, R. Lewanczuk, N. R. C. Campbell, D. Cooney and F. A. McAlister, "Randomized trial of the effect of pharmacist prescribing on improving blood pressure in the community: the Alberta clinical trial in optimizing hypertension (RxACTION)," *Circulation,* vol. 132, p. 93–100, 2015. |
| [41] | B. Bajorek, K. S. Lemay, P. Magin, C. Roberts, I. Krass and C. L. Armour, "Implementation and evaluation of a pharmacist-led hypertension management service in primary care: outcomes and methodological challenges," *Pharmacy Practice (Granada),* vol. 14, p. 0–0, 2016. |
| [42] | K. R. I. S. H. N. A. V. E. N. I. Kandasamy, A. G. I. L. A. N. Natarajan, J. O. Y. A. L. Sebastian, M. A. N. I. K. A. N. T. A. Konakalla, R. O. H. I. T. Sam, S. S. U. N. D. A. R. A. M. Rajagopal and S. A. M. B. A. T. H. K. U. M. A. R. RAMATHAN, "Impact of pharmacist intervention in screening and education on blood pressure in a rural area in Southern India," *Asian J Pharm Clin Res,* vol. 9, p. 339–43, 2016. |
| [43] | A. Y. Lam, J. K. Nguyen, J. J. Parks, D. E. Morisky, D. L. Berry and S. E. Wolpin, "Addressing low health literacy with “Talking Pill Bottles”: A pilot study in a community pharmacy setting," *Journal of the American Pharmacists Association,* vol. 57, p. 20–29, 2017. |
| [44] | M. Amer, N. U. Rahman, R. Nazir, S. Ur, A. Raza, H. Riaz, M. Sultana and S. Sadeeqa, "Impact of pharmacist's intervention on disease related knowledge, medication adherence, HRQoL and control of blood pressure among hypertensive patients.," *Pakistan journal of pharmaceutical sciences,* 2018. |
| [45] | E. Cheema, P. Sutcliffe, M. O. Weickert and D. R. J. Singer, "A randomised controlled trial of the impact of structured written and verbal advice by community pharmacists on improving hypertension education and control in patients with high blood pressure," *European journal of clinical pharmacology,* vol. 74, p. 1391–1395, 2018. |
| [46] | H. Okada, M. Onda, M. Shoji, N. Sakane, Y. Nakagawa, T. Sozu, Y. Kitajima, R. T. Tsuyuki and T. Nakayama, "Effects of lifestyle advice provided by pharmacists on blood pressure: The COMmunity Pharmacists ASSist for Blood Pressure (COMPASS-BP) randomized trial," *Bioscience trends,* vol. 11, p. 632–639, 2017. |
| [47] | D. M. Van der Laan, P. J. M. Elders, C. C. L. M. Boons, G. Nijpels, L. Van Dijk and J. G. Hugtenburg, "Effectiveness of a patient-tailored, pharmacist-led intervention program to enhance adherence to antihypertensive medication: the CATI study," *Frontiers in Pharmacology,* vol. 9, p. 1057, 2018. |
| [48] | N. Goruntla, V. Mallela and D. Nayakanti, "Effect of pharmacist directed counselling services on knowledge, attitude, and practice (kap) and blood pressure control in hypertensive patients: a randomized control trial," *Int J Pharm Sci Res,* vol. 10, p. 5109–16, 2019. |
| [49] | S. D. Alfian, J. F. M. van Boven, R. Abdulah, H. Sukandar, P. Denig and E. Hak, "Effectiveness of a targeted and tailored pharmacist-led intervention to improve adherence to antihypertensive drugs among patients with type 2 diabetes in Indonesia: A cluster randomised controlled trial," *British journal of clinical pharmacology,* vol. 87, p. 2032–2042, 2021. |
| [50] | K. R. Manigault, D. McKinley, S. Patel, C. Truong, S. Nguyen, A. Akil, L. Newsom, K. S. Murnane and M. M. Thurston, "The impact of a pharmacist-designed mobile application on blood pressure control and medication adherence in patients with hypertension," *Journal of the American College of Clinical Pharmacy,* vol. 3, p. 1286–1295, 2020. |
| [51] | P. Zhai, K. Hayat, W. Ji, Q. Li, L. Shi, N. Atif, S. Xu, P. Li, Q. Du and Y. Fang, "Efficacy of text messaging and personal consultation by pharmacy students among adults with hypertension: randomized controlled trial," *Journal of Medical Internet Research,* vol. 22, p. e16019, 2020. |
| [52] | I. L. Jackson and C. V. Ukwe, "Clinical outcomes of pharmaceutical care intervention in HIV positive patients with hypertension: a randomized controlled study," *Journal of Clinical Pharmacy and Therapeutics,* vol. 46, p. 1083–1094, 2021. |
| [53] | S. Khiali, N. Khezerlo-Aghdam, H. Namdar and T. Entezari-Maleki, "Pharmacist-Directed Self-Management of Blood Pressure Versus Conventional Management in Patients with Hypertension: A Randomized Control Trial," *High Blood Pressure & Cardiovascular Prevention,* vol. 28, p. 283–290, 2021. |
| [54] | Y. Li, G. Liu, C. Liu, X. Wang, Y. Chu, X. Li, W. Yang, Y. Shen, F. Wu and W. Zhang, "Effects of Pharmacist Intervention on Community Control of Hypertension: A Randomized Controlled Trial in Zunyi, China," *Global Health: Science and Practice,* vol. 9, p. 890–904, 2021. |
| [55] | Q. Liu, X. Zhu, M. Shen, J. Wu, S. Chen, Z. Wang, W. Yu, J. Shi, J. Huang and Z. Wang, "Community Pharmacist Services for Hypertensive Patients: A Novel Practice in Shanghai, China," *INQUIRY: The Journal of Health Care Organization, Provision, and Financing,* vol. 58, p. 00469580211020874, 2021. |
| [56] | N. Mehas, K. S. Hudmon, H. Jaynes, S. Klink, L. Downey and A. J. Zillich, "Impact of Electronic Medication Reminder Caps on Patient Adherence and Blood Pressure," *Journal of Pharmacy Technology,* vol. 37, p. 234–243, 2021. |
| [57] | A. Contreras-Vergara, S. Sifuentes-Franco, S. Haack, O. Graciano-Machuca, A. D. Rodriguez-Carrizalez, A. K. López-Contreras, I. V. Reyes-Pérez and S. G. Huerta-Olvera, "Impact of pharmaceutical education on medication adherence and its clinical efficacy in patients with type 2 diabetes and systemic arterial hypertension," *Patient preference and adherence,* p. 1999–2007, 2022. |
| [58] | A. Gupta, S. D. Ellis, C. Burkhardt, K. Young, D. R. Mazzotti, J. Mahnken, N. Abu-El-Rub, S. Chandaka, B. Comfort, D. Shanks and others, "Implementing a home-based virtual hypertension programme—a pilot feasibility study," *Family Practice,* vol. 40, p. 414–422, 2023. |
| [59] | M. Malik, A. Hussain, U. Aslam, A. Hashmi, M. Vaismoradi, K. Hayat and S. Jamshed, "Effectiveness of community pharmacy diabetes and hypertension care program: an unexplored opportunity for community pharmacists in Pakistan," *Frontiers in Pharmacology,* vol. 13, p. 710617, 2022. |
| [60] | A. S. A. R. A. H. MATHEWS and S. R. E. S. H. I. KUMARI, "IMPACT OF PHARMACIST LED HYPERTENSION MANAGEMENT," *Asian J Pharm Clin Res,* vol. 15, p. 23–26, 2022. |
| [61] | R. N. Okoro, I. Umate, J. D. Ohieku, S. I. Yakubu and M. O. Adibe, "The impact of pharmacist-led interventions on blood pressure control among patients with chronic kidney disease: A randomized controlled trial," *Journal of the American College of Clinical Pharmacy,* vol. 5, p. 1062–1074, 2022. |
| [62] | N. Paudel, S. Shrestha, N. R. Marasine, P. Khanal, S. Aryal, D. Erku and A. Poudel, "Impact of hospital pharmacist-delivered individualised pharmaceutical service intervention on clinical and patient-reported outcomes in patients with hypertension: a randomised controlled trial," *European Journal of Hospital Pharmacy,* vol. 30, p. 316–321, 2023. |
| [63] | M. Rohla, H. Haberfeld, M. Tscharre, K. Huber and T. W. Weiss, "Pharmacist interventions to improve blood pressure control in primary care: a cluster randomised trial," *International journal of clinical pharmacy,* vol. 45, p. 126–136, 2023. |
| [64] | A. Torres-Robles, S. I. Benrimoj, M. A. Gastelurrutia, F. Martinez-Martinez, T. Peiro, B. Perez-Escamilla, K. Rogers, I. Valverde-Merino, R. Varas-Doval and V. Garcia-Cardenas, "Effectiveness of a medication adherence management intervention in a community pharmacy setting: a cluster randomised controlled trial," *BMJ Quality & Safety,* vol. 31, p. 105–115, 2022. |
| [65] | W. Wang, L. Geng, C. Sun, H. Li, J. Wang and others, "Efficacy of pharmaceutical care in patients with type 2 diabetes mellitus and hypertension: a randomized controlled trial," *International Journal of Clinical Practice,* vol. 2022, 2022. |
| [66] | M. M. Gutierrez and R. Sakulbumrungsil, "Effectiveness of a pharmacist-led expert system intervention for medication adherence and blood pressure control of adults with hypertension: A randomized controlled trial," *Research in Social and Administrative Pharmacy,* vol. 19, p. 931–943, 2023. |
| [67] | R. Luque del Moral, M. A. Gastelurrutia, F. Martinez-Martinez, J. A. Jacomé, A. Dago, B. Suarez, N. Fikri-Benbrahim, M. Martı́, C. Nuñez, S. Sierra-Alarcón and others, "Effect of Pharmaceutical Intervention in Pharmacologically Treated Hypertensive Patients—A Cluster-Randomized Clinical Trial: AFPRES-CLM Study," *Journal of Personalized Medicine,* vol. 13, p. 1484, 2023. |
| [68] | P. M. Moreira, E. C. Aguiar, P. R. Castro, K. C. Almeida, J. A. Dourado, S. M. Paula, M. F. Melo, P. M. Santos and M. G. Oliveira, "Optimizing hypertension treatment in older patients through home blood pressure monitoring by pharmacists in primary care: the MINOR clinical trial," *Clinical Therapeutics,* vol. 45, p. 941–946, 2023. |
| [69] | I. E. Mozu, A. F. A. Marfo, J. S. Marfo, N. O. Adomako, N. K. Ayisi-Boateng, P. Boachie-Ansah, J. Attakorah and F. T. Owusu-Daaku, "Utilization of telepharmacy in the management of hypertension," *Exploratory Research in Clinical and Social Pharmacy,* vol. 12, p. 100381, 2023. |
| [70] | J. A. Earp, M. G. Ory and D. S. Strogatz, "The effects of family involvement and practitioner home visits on the control of hypertension.," *American Journal of Public Health,* vol. 72, p. 1146–1154, 1982. |
| [71] | P. E. Bogden, R. D. Abbott, P. Williamson, J. K. Onopa and L. M. Koontz, "Comparing standard care with a physician and pharmacist team approach for uncontrolled hypertension," *Journal of General Internal Medicine,* vol. 13, p. 740–745, 1998. |
| [72] | J. E. Borenstein, G. Graber, E. Saltiel, J. Wallace, S. Ryu, A. Jackson, S. Deutsch and S. R. Weingarten, "Physician-pharmacist comanagement of hypertension: a randomized, comparative trial," *Pharmacotherapy: The Journal of Human Pharmacology and Drug Therapy,* vol. 23, p. 209–216, 2003. |
| [73] | B. L. Carter, G. R. Bergus, J. D. Dawson, K. B. Farris, W. R. Doucette, E. A. Chrischilles and A. J. Hartz, "A cluster randomized trial to evaluate physician/pharmacist collaboration to improve blood pressure control," *The Journal of Clinical Hypertension,* vol. 10, p. 260–271, 2008. |
| [74] | J. S. Hunt, J. Siemienczuk, G. Pape, Y. Rozenfeld, J. MacKay, B. H. LeBlanc and D. Touchette, "A randomized controlled trial of team-based care: impact of physician-pharmacist collaboration on uncontrolled hypertension," *Journal of general internal medicine,* vol. 23, p. 1966–1972, 2008. |
| [75] | D. L. McLean, F. A. McAlister, J. A. Johnson, K. M. King, M. J. Makowsky, C. A. Jones, R. T. Tsuyuki, S. C. R. I. P.-H. T. N. Investigators and others, "A randomized trial of the effect of community pharmacist and nurse care on improving blood pressure management in patients with diabetes mellitus: Study of Cardiovascular Risk Intervention by Pharmacists–Hypertension (SCRIP-HTN)," *Archives of internal medicine,* vol. 168, p. 2355–2361, 2008. |
| [76] | V. Santschi, N. Rodondi, O. Bugnon and M. Burnier, "Impact of electronic monitoring of drug adherence on blood pressure control in primary care: a cluster 12-month randomised controlled study," *European journal of internal medicine,* vol. 19, p. 427–434, 2008. |
| [77] | B. L. Carter, G. Ardery, J. D. Dawson, P. A. James, G. R. Bergus, W. R. Doucette, E. A. Chrischilles, C. L. Franciscus and Y. Xu, "Physician and pharmacist collaboration to improve blood pressure control," *Archives of internal medicine,* vol. 169, p. 1996–2002, 2009. |
| [78] | S. Rinfret, M.-T. Lussier, A. Peirce, F. Duhamel, S. Cossette, L. Lalonde, C. Tremblay, M.-C. Guertin, J. LeLorier, J. Turgeon and others, "The impact of a multidisciplinary information technology–supported program on blood pressure control in primary care," *Circulation: Cardiovascular Quality and Outcomes,* vol. 2, p. 170–177, 2009. |
| [79] | D. Edelman, S. K. Fredrickson, S. D. Melnyk, C. J. Coffman, A. S. Jeffreys, S. Datta, G. L. Jackson, A. C. Harris, N. S. Hamilton, H. Stewart and others, "Medical clinics versus usual care for patients with both diabetes and hypertension: a randomized trial," *Annals of internal medicine,* vol. 152, p. 689–696, 2010. |
| [80] | H. Tobari, T. Arimoto, N. Shimojo, K. Yuhara, H. Noda, K. Yamagishi and H. Iso, "Physician–pharmacist cooperation program for blood pressure control in patients with hypertension: a randomized-controlled trial," *American journal of hypertension,* vol. 23, p. 1144–1152, 2010. |
| [81] | C. A. Weber, M. E. Ernst, G. S. Sezate, S. Zheng and B. L. Carter, "Pharmacist-physician comanagement of hypertension and reduction in 24-hour ambulatory blood pressures," *Archives of internal medicine,* vol. 170, p. 1634–1639, 2010. |
| [82] | A. M. Albsoul-Younes, E. A. Hammad, N. A. Yasein and L. M. Tahaineh, "Pharmacist-physician collaboration improves blood pressure control.," *Saudi medical journal,* vol. 32, p. 288–292, 2011. |
| [83] | L. B. Cohen, T. H. Taveira, S. A. M. Khatana, A. G. Dooley, P. A. Pirraglia and W.-C. Wu, "Pharmacist-led shared medical appointments for multiple cardiovascular risk reduction in patients with type 2 diabetes," *The Diabetes Educator,* vol. 37, p. 801–812, 2011. |
| [84] | V. Santschi, A. Lord, D. Berbiche, D. Lamarre, L. Corneille, L. Prud'homme, M. Normandeau and L. Lalonde, "Impact of collaborative and multidisciplinary care on management of hypertension in chronic kidney disease outpatients," *Journal of Pharmaceutical Health Services Research,* vol. 2, p. 79–87, 2011. |
| [85] | B. L. Svarstad, J. M. Kotchen, T. I. Shireman, R. L. Brown, S. Y. Crawford, J. K. Mount, P. A. Palmer, E. M. Vivian and D. A. Wilson, "Improving refill adherence and hypertension control in black patients: Wisconsin TEAM trial," *Journal of the American Pharmacists Association,* vol. 53, p. 520–529, 2013. |
| [86] | J. D. Hirsch, N. Steers, D. S. Adler, G. M. Kuo, C. M. Morello, M. Lang, R. F. Singh, Y. Wood, R. M. Kaplan and C. M. Mangione, "Primary care–based, pharmacist–physician collaborative medication-therapy management of hypertension: a randomized, pragmatic trial," *Clinical therapeutics,* vol. 36, p. 1244–1254, 2014. |
| [87] | A. Leiva, A. Aguilo, M. Fajo-Pascual, L. Moreno, M. C. Martı́n, E. M. Garcia, R. E. Duro, F. Serra, P. Dagosto, A. A. Iglesias-Iglesias and others, "Efficacy of a brief multifactorial adherence-based intervention in reducing blood pressure: a randomized clinical trial," *Patient preference and adherence,* p. 1683–1690, 2014. |
| [88] | B. L. Carter, C. S. Coffey, G. Ardery, L. Uribe, D. Ecklund, P. James, B. Egan, M. V. Weg, E. Chrischilles and T. Vaughn, "Cluster-randomized trial of a physician/pharmacist collaborative model to improve blood pressure control," *Circulation: Cardiovascular Quality and Outcomes,* vol. 8, p. 235–243, 2015. |
| [89] | B. Qudah, A. Albsoul-Younes, E. Alawa and N. Mehyar, "Role of clinical pharmacist in the management of blood pressure in dialysis patients," *International journal of clinical pharmacy,* vol. 38, p. 931–940, 2016. |
| [90] | D. Scala, E. Menditto, G. Caruso, V. M. Monetti, V. Orlando, F. Guerriero, G. Buonomo, D. Caruso and M. D’Avino, "Are you more concerned about or relieved by medicines? An explorative randomized study of the impact of telephone counseling by pharmacists on patients’ beliefs regarding medicines and blood pressure control," *Patient education and counseling,* vol. 101, p. 679–686, 2018. |
| [91] | C. A. Peralta, M. Frigaard, L. Rolon, K. Seal, D. Tuot, J. Senyak, L. Lo, N. Powe, R. Scherzer, S. Chao and others, "Screening for CKD to improve processes of care among nondiabetic veterans with hypertension: a pragmatic cluster-randomized trial," *Clinical Journal of the American Society of Nephrology: CJASN,* vol. 15, p. 174, 2020. |
| [92] | V. Santschi, G. Wuerzner, B. Pais, A. Chiolero, P. Schaller, L. Cloutier, G. Paradis and M. Burnier, "Team-Based Care for Improving Hypertension Management: A Pragmatic Randomized Controlled Trial," *Frontiers in cardiovascular medicine,* vol. 8, 2021. |
| [93] | D. Lau, J. Ringrose, F. A. McAlister, M. Fradette, P. W. Wood, P. Boulanger, S. Klarenbach, J. M. Holroyd-Leduc, K. Alagiakrishnan, D. Rabi and others, "Telemonitoring and protocolized case management for hypertensive community dwelling older adults (TECHNOMED): a randomized controlled trial," *Journal of Hypertension,* vol. 40, p. 1702–1712, 2022. |
| [94] | I. A. Naqvi, K. Strobino, Y. Kuen Cheung, H. Li, K. Schmitt, S. Ferrara, S. E. Tom, A. Arcia, O. A. Williams, I. M. Kronish and others, "Telehealth after stroke care pilot randomized trial of home blood pressure telemonitoring in an underserved setting," *Stroke,* vol. 53, p. 3538–3547, 2022. |
| [95] | M. N. Nguyen-Huynh, J. D. Young, B. Ovbiagele, J. G. Alexander, S. Alexeeff, C. Lee, N. Blick, B. J. Caan, A. S. Go and S. Sidney, "Effect of lifestyle coaching or enhanced pharmacotherapy on blood pressure control among Black adults with persistent uncontrolled hypertension: a cluster randomized clinical trial," *JAMA Network Open,* vol. 5, p. e2212397–e2212397, 2022. |
| [96] | L. A. McGuinness and J. P. T. Higgins, "Risk-of-bias VISualization (robvis): An R package and Shiny web app for visualizing risk-of-bias assessments," *Research Synthesis Methods,* vol. n/a. |
| [97] | J. A. C. Sterne, J. Savović, M. J. Page, R. G. Elbers, N. S. Blencowe, I. Boutron, C. J. Cates, H.-Y. Cheng, M. S. Corbett, S. M. Eldridge and others, "RoB 2: a revised tool for assessing risk of bias in randomised trials," *bmj,* vol. 366, 2019. |
| [98] | N. R. Haddaway, M. J. Page, C. C. Pritchard and L. A. McGuinness, "PRISMA2020: An R package and Shiny app for producing PRISMA 2020-compliant flow diagrams, with interactivity for optimised digital transparency and Open Synthesis," *Campbell Systematic Reviews,* vol. 18, p. e1230, 2022. |
